# Long forgotten: *Eunice woodwardi* Baird, 1869 (Annelida, Eunicidae) revisited, with an insight on internal anatomy

**DOI:** 10.7717/peerj.13126

**Published:** 2022-04-14

**Authors:** María Barroso, Juan Moreira, Julio Parapar

**Affiliations:** 1 Departamento de Bioloxía, Universidade da Coruña, A Coruña, Spain; 2 Departamento de Biología (Zoología) & Centro de Investigación en Biodiversidad y Cambio Global (CIBC-UAM), Universidad Autónoma de Madrid, Madrid, Spain

**Keywords:** Eunicidae, Anatomy, Iberian peninsula, SEM, Micro-CT, Distribution

## Abstract

*Eunice woodwardi*Baird, 1869, originally described from the Ría de A Coruña (NW Iberian Peninsula), has been overlooked and never reported from the Atlantic coast of Spain after original description and the subsequent redescription of the holotype. In the present study, we revised comparatively the holotype, newly collected specimens of this species and specimens identified as *Eunice vittata* (Delle Chiaje, 1829) from western Mediterranean Sea. The validity of *E. woodwardi* is supported and previous descriptions are complemented after a throughout study of the external morphology by means of light compound microscopy and scanning electron microscopy, and that of the internal anatomy by histological sectioning and micro-computed tomography. The presence of eyes, nuchal organs, dorsal and ventral ciliary organs on parapodial cirri and paired nephridia in most segments is confirmed in *E. woodwardi*; the digestive tract is clearly regionalized and divided into pharynx, oesophagus, stomach, fore, mid- and hind intestine. The presence of *E. woodwardi* in the Ría de Ferrol is also reported, and we suggest that previous records of *E. vittata* in NW Iberian Peninsula should be reviewed. *Eunice woodwardi* is distinguished by a set of characters such as having non-articulated and non-constricted cephalic appendages, the maxillary formula, the range of branchial distribution, maximum number of branchial filaments, number of limbate and compound falciger chaetae per parapodium, the presence of an apical mucro in the guard of falciger chaetae blades and the number of teeth in pectinate chaetae. Epibiont Ciliophora on branchiae are also reported.

## Introduction

*Eunice* Cuvier, 1817 is the most species-rich genus of the annelid family Eunicidae Berthold, 1827 and comprises 250 valid species (Read & Fauchald, 2022). It is widely distributed and can be found in soft and hard substrates from the intertidal zone to the deep sea in all oceans (Hutchings, 1986). The diagnosis of the genus was traditionally based on a combination of several morphological characters, such as the presence of three prostomial antennae, a pair of palps, a pair of peristomial cirri, and a set of chaetal types that includes: limbate, pectinate and compound chaetae, and subacicular hooks (Orrhage, 1995). However, all these are plesiomorphic characters shared with other eunicid genera such as *Euniphysa* Wesenberg-Lund, 1949 (Lu & Fauchald, 2000) and even with members of other families such as Onuphidae Kinberg, 1865 (Zanol, Fauchald & Paiva, 2007). Furthermore, original descriptions of many *Eunice* species are often based on incomplete or a low number of specimens and therefore intraspecific variability is not well delimited. Consequently, many species are not characterized properly and this represents the main issue in assessing the true diversity of the genus (Miura, 1986; Fauchald, 1992). In this context, species of *Eunice* had traditionally been “informally” grouped according to characters lacking real systematic value, such as the branchial distribution patterns and the colour and teeth number of subacicular hooks (*e.g*., Ehlers, 1868; Hartman, 1944; Fauchald, 1970; Carrera-Parra & Salazar-Vallejo, 1998). In fact, recent phylogenetic studies on Eunicidae conclude that *Eunice *sensu* lato* is actually non-monophyletic (Zanol et al., 2010, 2021; Zanol, Halanych & Fauchald, 2014). Besides, the genus *Leodice* has recently been resurrected and several species of *Eunice* transferred there (Zanol, Halanych & Fauchald, 2014). Zanol et al. (2021) also suggest that some *Eunice* species correspond to a clade defined by branchiae starting late, ventral cirri with inflated base shaped as thick ventral ridges, left MxIV with dorsal teeth only, and anteriormost chaetigers bearing bidentate falciger chaetae with inconspicuous blade teeth. However, there is no consensus in placement of some species and whether there are reliable morphological characters to clearly delineate the involved genera (Zanol, Halanych & Fauchald, 2014; Molina-Acevedo & Carrera-Parra, 2017). Therefore, it seems that further phylogenetic analyses based in a wide sampling of species and molecular data are needed to clarify the actual systematics of species included in *Eunice* and *Leodice* (Zanol et al., 2021).

On the other hand, the general internal anatomy (*e.g*., nervous system, muscular system, circulatory system and nephridia) of a number of annelid families is well known (see Bartolomaeus & Purschke, 2005; Zanol, 2010), but only Ehlers (1868) studied in detail the digestive tract of the genus *Eunice* as well as other Eunicidae. Nevertheless, many anatomical features of the digestive tract of this genus still remain undescribed.

Eight species of *Eunice *sensu* lato* have been reported in the Iberian Peninsula: *E. norvegica* (Linnaeus, 1767), *E. pennata* (Müller, 1776), *E. vittata* (Delle Chiaje, 1829), *E. purpurea* Grube, 1866, *E. roussaei* Quatrefages, 1866, *E. woodwardi*Baird, 1869, *E. schizobranchia* Claparède, 1870 and *E. dubitata* Fauchald, 1974. *Eunice woodwardi* was collected by MacAndrew & Woodward (1864) during benthic sampling in the Ría de A Coruña (NW Spain). This species is morphologically very close to *E. vittata* mostly regarding the presence of tridentate subacicular hooks, that is a highly relevant diagnostic character. However, *E. woodwardi* has been largely ignored and overlooked after original description; for instance, later monographies on European polychaetes (*e.g.*, Fauvel, 1923) do not even mention this species. Hartman (1959) questioned whether *E. woodwardi* was a synonym of *E. vittata*, and finally regarded it as indeterminable. Later, Fauchald (1992) re-examined the holotype of *E. woodwardi* and consider it as a valid species, also providing a redescription. Nevertheless, several key characters in *Eunice*, such as the maxillary formula were not described since this would imply dissection and consequently a potential deterioration of the only specimen available. Anyway, it seems that *E. woodwardi* fell into oblivion despite Fauchald’s redescription. This might also be due to *E. vittata* being considered as a cosmopolitan species and, therefore, it is likely that specimens of *Eunice* with tridentate subacicular hooks might have often been identified as *E. vittata* across the world. In fact, the presence of *E. woodwardi* in NW Spain also remained overlooked even in recent publications (*e.g*., Parapar et al., 1993; Parapar, Urgorri & Besteiro, 1996; Parapar & Moreira, 2009; Besteiro et al., 2018). In this context, Zanol et al. (2021) challenge the wide range of distribution traditionally attributed to several eunicids, suggesting that *E. vittata* might have a more restricted distribution.

In this study, we present an integrative approach to the taxonomy and anatomy of *E. woodwardi* in order to support the validity of the taxon. Additional anatomical information is provided after examination of the holotype and non-type material collected near the type locality; the original description and redescription were complemented with other characters not observed in the holotype, also assessing its intraspecific variability. The main features of the internal anatomy of *E. woodwardi* are described for the first time and the presence of epibiont ciliophorans on branchiae is also reported.

## Materials and Methods

This work is based on the study of the holotype of *E. woodwardi* from off A Coruña (Galicia, NW Spain), along with 34 newly collected specimens collected in the nearby Ría de Ferrol (Table 1). Selected specimens have been deposited in the Museo Nacional de Ciencias Naturales, Madrid, Spain (MNCN) and the British Museum (Natural History), London, England (NHML). For comparative purposes, specimens identified as *E. vittata* from several Mediterranean locations were also examined: Two specimens from Naples, Italy (type locality; Museum of Natural History of Wroclaw, MNHW), five from Venice, Italy (Zoological Museum Hamburg, ZMH), two from Banyuls-sur-Mer, France (ZMH), five from Valencia, Spain (MNCN) and four from Mallorca, Spain. Type material could not be examined because there are no longer in existence (Fauchald, 1992, p. 337). The description of *E. woodwardi* holotype is based on Fauchald (1992) and is complemented with our observations. Additional observations of material collected in the Ría de Ferrol and *E. vittata* from Naples are also provided.

**Table 1:** Collection data, voucher numbers and examination techniques of specimens of *Eunice woodwardi* and ­specimens identified as *E. vittata* studied in this work.

Species/Locality	Site	Voucher number	Date	Latitude	Longitude	Depth (m)	Habitat	Examination technique	Figures
***Eunice woodwardi***									
A Coruña	–	ZH 1863.8.19.13 (Holotype)	1863	N/A	N/A	N/A	N/A	LCM	1A–1D
Ferrol	71	MNCN 16.01/19142	30/03/1987	43°29′16″	08°10′44″	1.5	Muddy sand	Micro-CT	
"	A Malata	MNCN 16.01/19143	18/02/1987	43°29′27″	08°14′48″	3	"	"	12A–12F
"	25B	MNCN 16.01/19144	12/09/1987	43°28′08″	08°15′37″	13	Gravel	SEM	5E, 5F, 9E, 18C
"	41	MNCN 16.01/19145	06/09/1987	43°28′10″	08°13′44″	"	Muddy sand	LCM	
"	"	MNCN 16.01/19146	"	"	"	"	"	SEM	
"	"	MNCN 16.01/19147	"	"	"	"	"	HIS	13A–13F, 15B, 15D, 16A–16D, 18A, 18B
"	"	MNCN 16.01/19148	"	"	"	"	"	"	11A, 11B, 16E
"	"	MNCN 16.01/19149	"	"	"	"	"	Micro-CT	10A–10D, 11C, 11D, 15A
"	"	MNCN 16.01/19150	"	"	"	"	"	LCM	8G, 8H
"	21	MNCN 16.01/19151	08/08/1987	43°28′10″	08°14′47″	8	"	"	
"	38	MNCN 16.01/19152	18/07/1987	43°27′30″	08°13′44″	10	"	"	7A, 7D
"	"	MNCN 16.01/19153	"	"	"	"	"	SEM	8F
"	63	MNCN 16.01/19154	25/08/1987	43°28′31″	08°13′44″	5	Sandy mud	LCM	
"	"	MNCN 16.01/19155	"	"	"	"	"	SEM	5A, 5B, 8E, 9D
"	"	MNCN 16.01/19156	"	"	"	"	"	"	6A, 8I, 19A–19D
"	"	MNCN 16.01/19157	"	"	"	"	"	FESEM	17E, 17F
"	"	MNCN 16.01/19158	"	"	"	"	"	SEM	9A, 9C, 17A
"	68	MNCN 16.01/19159	30/03/1987	43°28′51″	08°11′13″	"	Muddy sand	LCM	4B–4D, 7B, 7C, 7E–7H
		MNCN 16.01/19160	"	"	"	"	"	HIS	17G
"	"	MNCN 16.01/19161	"	"	"	"	"	SEM	
"	36	MNCN 16.01/19162	06/09/1987	43°28′25″	08°14′47″	10	"	LCM	
"	"	MNCN 16.01/19163	"	"	"	"	"	SEM	5C, 5D, 8A–8D, 8J, 9B
"	17	MNCN 16.01/19164	28/06/2010	43°27′42″	08°17′05″	26.5	Muddy sandy gravel	SEM	6A, 14A–14E, 15C
"	26A	MNCN 16.01/19165	29/06/2010	43°27′39″	08°15′58″	9.3	Gravelly mud	LCM	
"	"	MNCN 16.01/19166	"	"	"	"	"	FESEM	6B, 6C, 16F, 16G, 17B–17D
"	–	MNCN 16.01/19167	09/05/2019	43°27′43″	08°15′39″	20	Muddy sand	LCM	4B
"	–	MNCN 16.01/19168	17/03/2021	43°27′44″	08°16′29″	15	"	"	4A
"	–	MNCN 16.01/19169	26/05/2021	43°27′52″	08°16′55″	8	Mud	"	2A–2E, 3A–3D
***Eunice vittata***									
Naples	–	–	1900	N/A	N/A	N/A	N/A	LCM	20A–20D
Venice	–	ZMH-V 12932	1961	"	"	"	"	"	
Banyuls-sur-Mer	–	ZMH-P 14276	N/A	"	"	"	"	"	
Valencia	–	MNCN 16.01/2677	"	"	"	"	"	"	
"	–	MNCN 16.01/2723	"	"	"	"	"	"	
Mallorca	–	SPR04-03	24/07/2017	"	"	"	Gravel	"	
"	–	PAD04-03	"	"	"	"	Muddy sand	"	
"	–	CBR01-06	25/07/2017	"	"	"	Gravel	"	
"	–	CBR01-11	"	"	"	"	"	"	

**Note:**

FESEM, Field Emission Scanning Electron Microscope; HIS, Histological Sectioning; LCM, Light Compound Microscopy; micro-CT, micro-computed X-ray tomography; SEM, Scanning Electron Microscopy.

### Sample collection

Specimens previously identified as *E. vitatta* by Parapar et al. (1993) were collected in 1988 and 1989 in the Ría de Ferrol: (1) directly by hand in the rocky intertidal, (2) by pushing PVC corers into subtidal soft sediment and (3) by means of a Naturalist dredge deployed on subtidal sedimentary bottoms (see Parapar et al., 1993 for details). Additional samplings were done in subtidal soft bottoms at the Ría de Ferrol with a Van Veen grab in 2010, 2019 and 2021. Specimens were sorted from samples, fixed in 4% formalin for 24–48 h and subsequently transferred to 70% ethanol for preservation.

### Light microscopy, SEM and FESEM

External anatomy of *E. woodwardi* was studied using an Olympus SZX12 stereomicroscope and an Olympus BX51 light compound microscope connected to a drawing tube. Specimens used for examination with Scanning Electron Microscope (SEM), were dehydrated *via* a graded ethanol series, critical point dried, coated with gold in a BAL-TEC SCD 004 evaporator, and examined and photographed under a JEOL JSM-6400 at the Servizos de Apoio á Investigación (SAI, Universidade da Coruña) (Parapar et al., 2017). Those used for examination with Field Emission Scanning Electron Microscope (FESEM) were instead cleaned by ultrasound, *via* a graded ethanol series, critical point dried, coated with iridium in a BAL-TEC SCD 004 evaporator, and examined and photographed under a JEOL JSM-7200F also at the SAI.

### Anatomical study

Two specimens preserved in 70% ethanol were used for histological sectioning; they were dehydrated through a series of graded ethanol baths and clearing agent, infiltrated with paraffin and xylene in 1:1 proportion at 57 °C overnight and embedded in a paraffin block. The block was sectioned with a microtome in 8 μm sections, which were placed on microscope slides, hydrated, and stained with haematoxylin–eosin, dehydrated and finally mounted on permanent slides with Canada balsam.

Specimens studied with micro-computed X-ray tomography (micro-CT hereafter) were originally preserved in 70% ethanol and dehydrated in successive baths of ethanol 90% and 96%, then immersed 2 h in hexamethyldisilazane and allowed to air dry overnight (Alba-Tercedor & Sánchez-Tocino, 2011; Parapar et al., 2019) at the Estación de Bioloxía Mariña da Graña, Universidade de Santiago de Compostela, Spain (REBUSC-EBMG, USC). No staining was used. Scanning was carried out with a microtomograph Skyscan 1172 using the following parameters: 55 kV, 165 mA, unfiltered, image pixel size of 3.94 and 6.78 μm and no camera binning. Images were treated with Skyscan software: they were reconstructed with the NRecon software and cleaned with CT Analyzer software; to visualize the data, DataViewer and CTVox softwares were used. Datasets of transverse 2D images of studied specimens were uploaded at the Morphosource repository (https://www.morphosource.org/catalog/media?utf8=%E2%9C%93&locale=en&search_field=all_fields&q=eunice+woodwardi).

## Results

***Eunice woodwardi* Baird, 1869**

*Eunice woodwardi*Baird, 1869: 347; Fauchald, 1992: 343–345, Fig. 117, Tables 41–42.

*Eunice vittata* (Delle Chiaje). Parapar et al., 1993: 117–125 (non Delle Chiaje, 1829).

**Material examined**

Type material: *Eunice woodwardi*Baird, 1869. Holotype (BM(NH) ZH 1863.8.19.13; Ría de A Coruña; Table 1).

Non-type material: 34 specimens (MNCN 16.01/19142 to 16.01/19169; Ría de Ferrol; Table 1).

**External morphology**

**Holotype**

**Figure 1:**
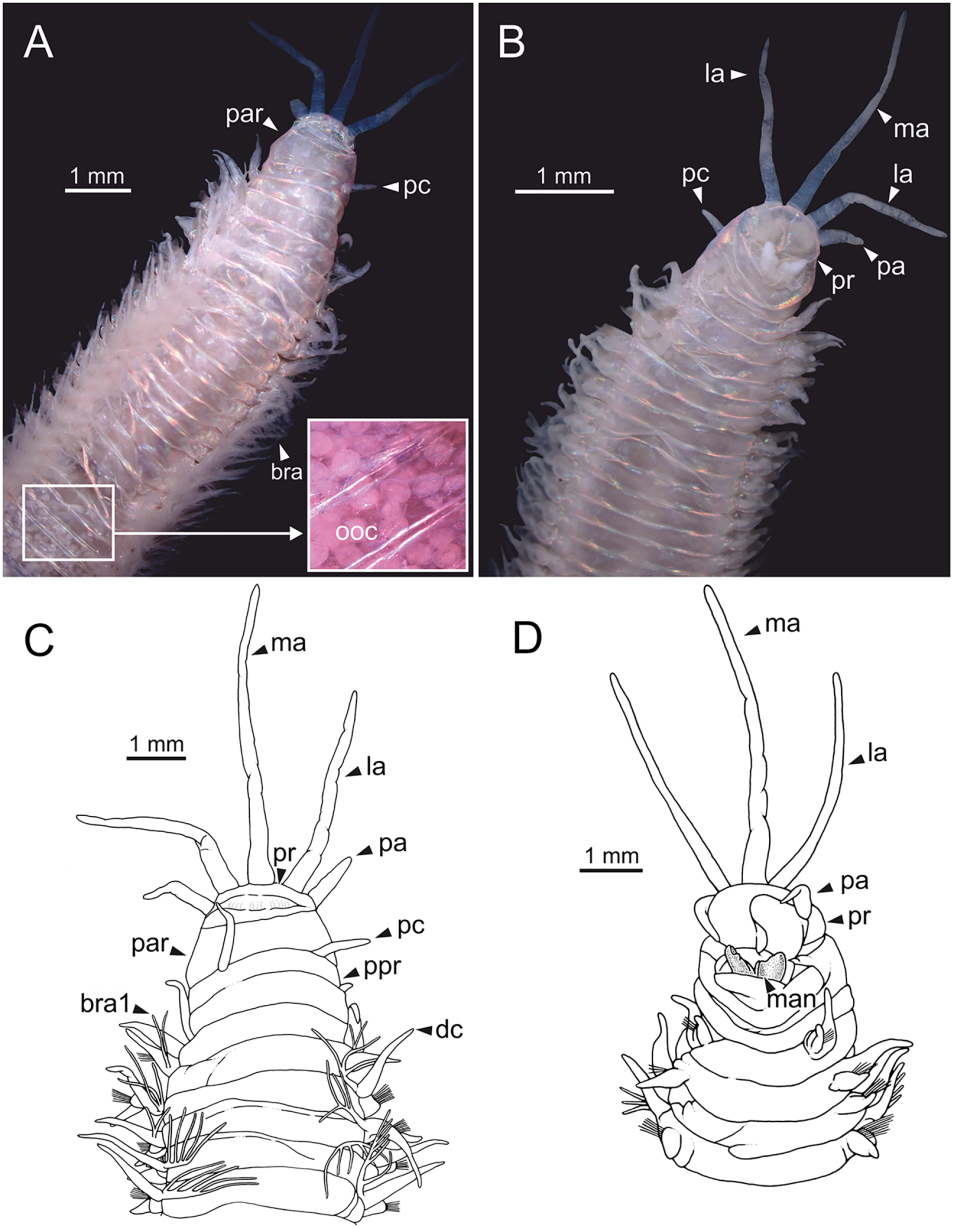
Stereomicrographs and line drawings of *Eunice woodwardi*. Holotype (BM(NH) ZH 1863.8.19.13). (A, B) Anterior end in dorsal and ventral view. (C, D) Line drawings of anterior end in dorsal (C) and ventral view (D). bra, branchia; bra1, branchia 1; dc, dorsal cirrus; la, lateral antenna; ma, median antenna; man, mandibles, ooc, oocytes; pa, palp, par, peristomial anterior ring; pc, peristomial cirrus; ppr, peristomial posterior ring; pr, prostomium.

Incomplete specimen, 49 mm long, 5 mm wide, with 59 chaetigers. Mature female, oocytes first observed in chaetiger 18. Body slightly flattened dorsoventrally, whitish (Figs. 1A, 1B); original colouration not preserved.

Prostomium nearly as wide as peristomium but shorter (Fig. 1D). Prostomial lobes frontally rounded and obliquely divided (Figs. 1B, 1D). Prostomial appendages equally separated from each other and arranged in horseshoe shape; all antennae with ring-shaped ceratophores; ceratostyles slender, digitiform, smooth, lacking distinct articulations or constrictions (Figs. 1A–1D). Median antenna about 1.2 times as long as lateral antennae; palps one third as long as lateral antennae (Figs. 1A–1D). Eyes not seen. Peristomium cylindrical; anterior ring longer than posterior one (Fig. 1C); peristomial cirri digitiform, smooth, slightly thinner than palps (Fig. 1C). Maxillary apparatus and mandibles not observed; mandible distal end just protruding from oral cavity (Fig. 1D).

Branchiae pectinate, branchial stem longer than dorsal cirri (Fig. 1C), present from chaetiger 3 to 39; first branchia with two filaments (Fig. 1C); up to 12 filaments per branchia in following chaetigers. Chaetigers 15–24 showing maximum number of filaments; last chaetigers of branchial region with 1–2 filaments. Branchial filaments of mid-branchial region longer or about as long as dorsal cirri.

Parapodia sub-biramous. Dorsal cirri smooth, digitiform, tapering. Ventral cirri short with a digitiform tip, inflated basally from chaetiger 3 to 40. Dorsal and ventral cirri decreasing in length from anterior to posterior parapodia (Table S1).

Pre- and postchaetal lobes low, transverse folds. Prechaetal lobes folds covering bases of compound falcigers. Acicular lobes truncate. 3–4 notoaciculae, thin, distally bent; two (sometimes three) yellow neuroaciculae, one larger than the other, tapering with blunt tips, curved distally and protruding from acicular lobe, never T-shaped. Chaetae including 5–13 limbate, 1–3 pectinate, 5–20 compound falcigers and 1–5 tridentate subacicular hooks. Limbate and pectinate chaetae arranged in a bundle dorsal to neuroaciculae. Limbate chaetae elongated, marginally serrated, distally curved and tapering. All pectinate chaetae heterodont, about 0.3–0.4 times as long as limbate chaetae; one external tooth three times as long as others. Compound falcigers ventral to neuroaciculae; shafts distally inflated and marginally serrated. Blades bidentate; proximal tooth slightly larger than distal tooth, triangular, perpendicular to blade axis; distal tooth curved dorsally; distal end protected by elongated guard, marginally serrated and provided with conspicuous apical mucro. Number of limbate and compound falcigers decreasing from anterior to posterior chaetigers (Tables S2, S3). Subacicular hooks from chaetiger 31, yellowish, tridentate with teeth in a crest, distal end protected by rounded guards. Number of hooks increasing from 1 to 5 from chaetiger 31 to 59. Hooks almost hidden within parapodia in anterior chaetigers and protruding conspicuously in last chaetigers.

**Non-type material**

Largest complete specimen 75 mm long, 4 mm wide, with 112 chaetigers; mean length 30 mm (standard deviation, S.D. = 18), mean width 1.2 mm (S.D. = 0.9 mm), with 37–112 chaetigers.

**Figure 2:**
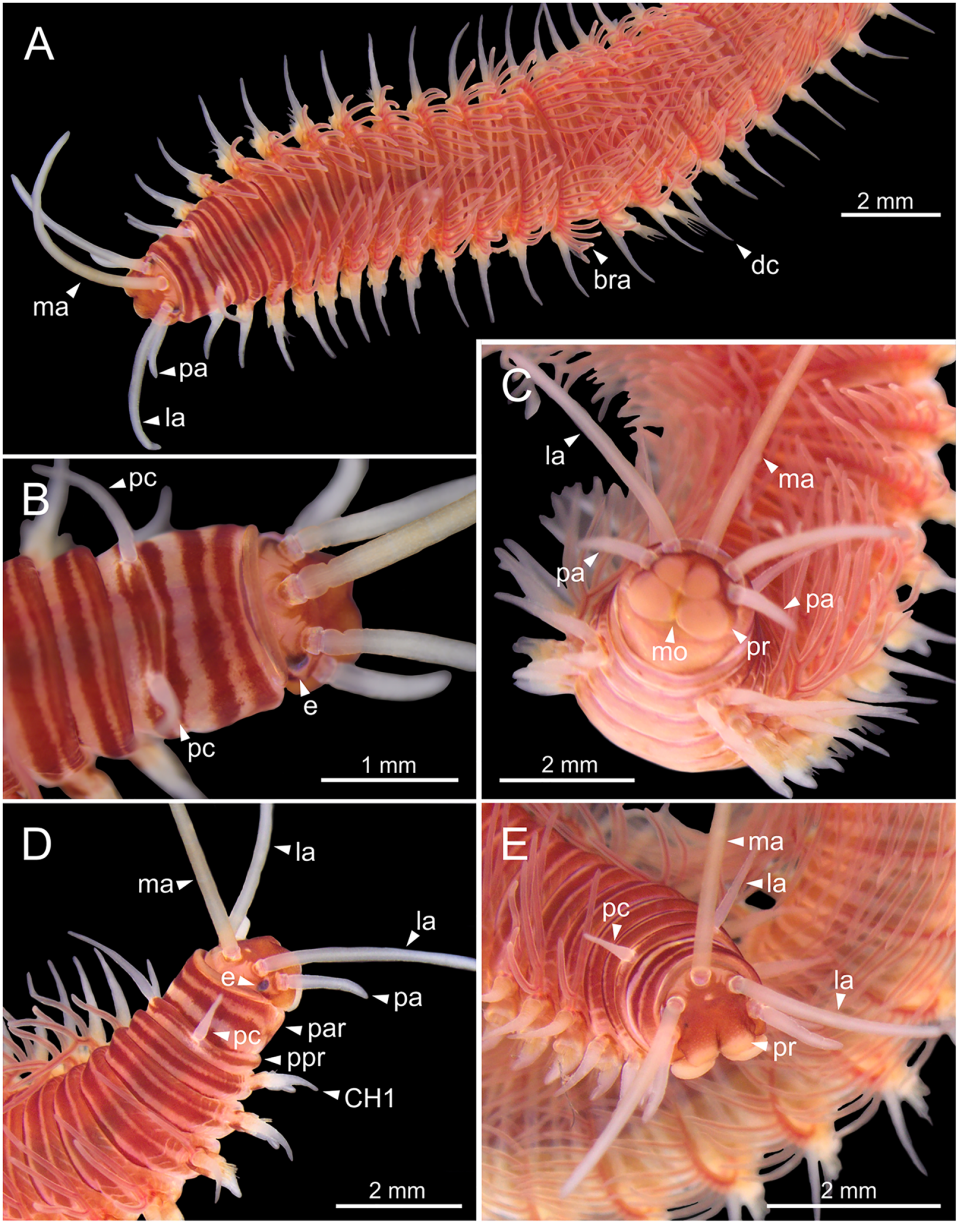
Stereomicrographs of *Eunice woodwardi*. One alive specimen (Ría de Ferrol, MNCN 16.01/19169). (A) Anterior end, dorsal view; (B) anterior end, dorsal view; (C) anterior end, frontal view; (D) anterior end and first chaetigers, dorso-lateral view; (E) anterior end and first chaetigers, dorso-frontal view. bra, branchia; CH1, chaetiger 1; dc, dorsal cirrus; e, eye; la, lateral antenna; ma, median antenna; mo, mouth; pa, palp; par, peristomial anterior ring; pc, peristomial cirrus; ppr, peristomial posterior ring; pr, prostomium.

**Figure 3:**
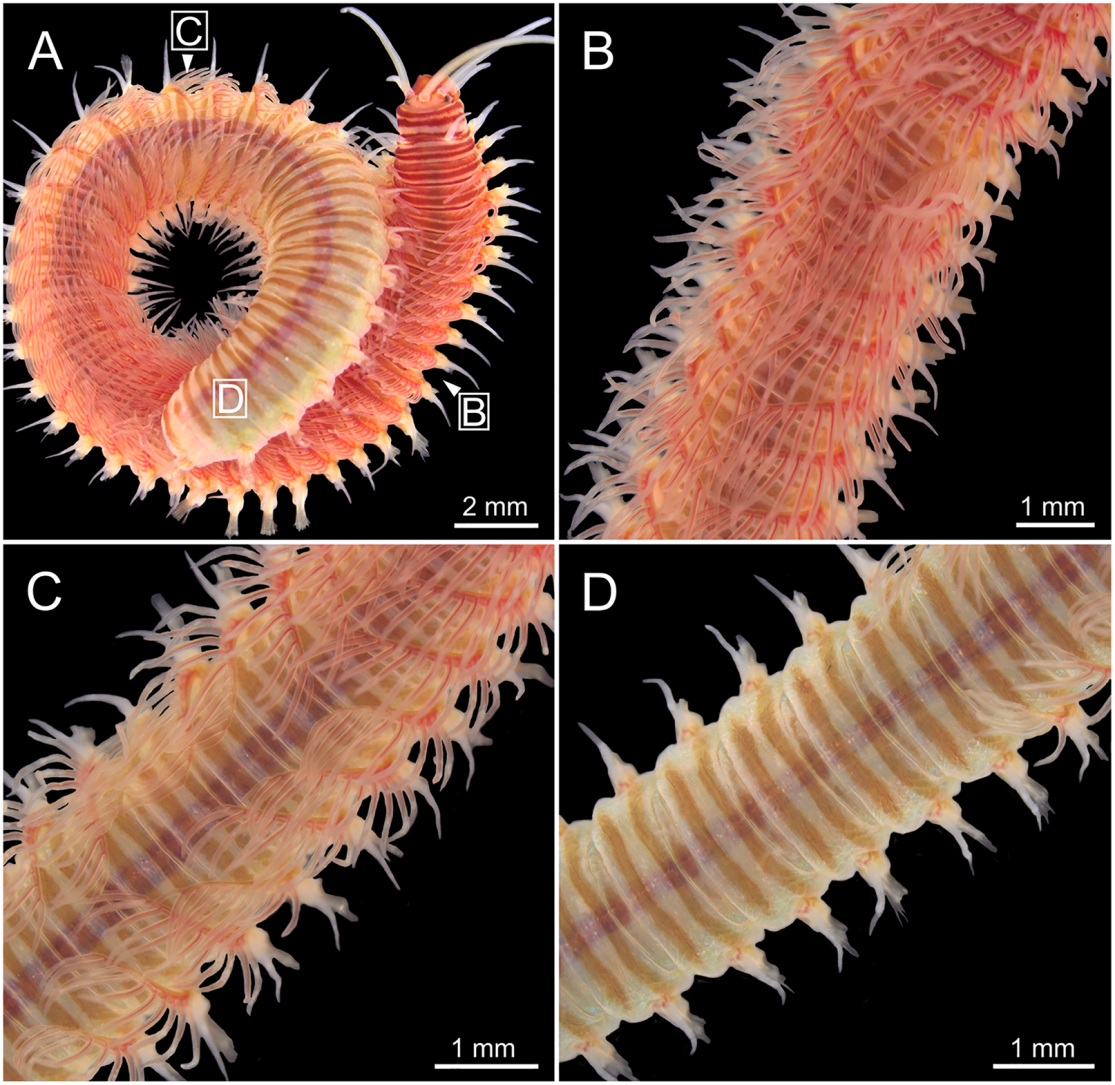
Stereomicrographs of *Eunice woodwardi*. One alive specimen (Ría de Ferrol, MNCN 16.01/19169). (A) Full specimen, dorsal view; (B) chaetigers of anterior branchial region, dorsal view; (C) chaetigers of posterior branchial region, dorsal view; (D) abranchiate chaetigers of posterior body half, dorsal view. Framed capital letters in (A) refer to figure parts (B) to (D).

**Figure 4:**
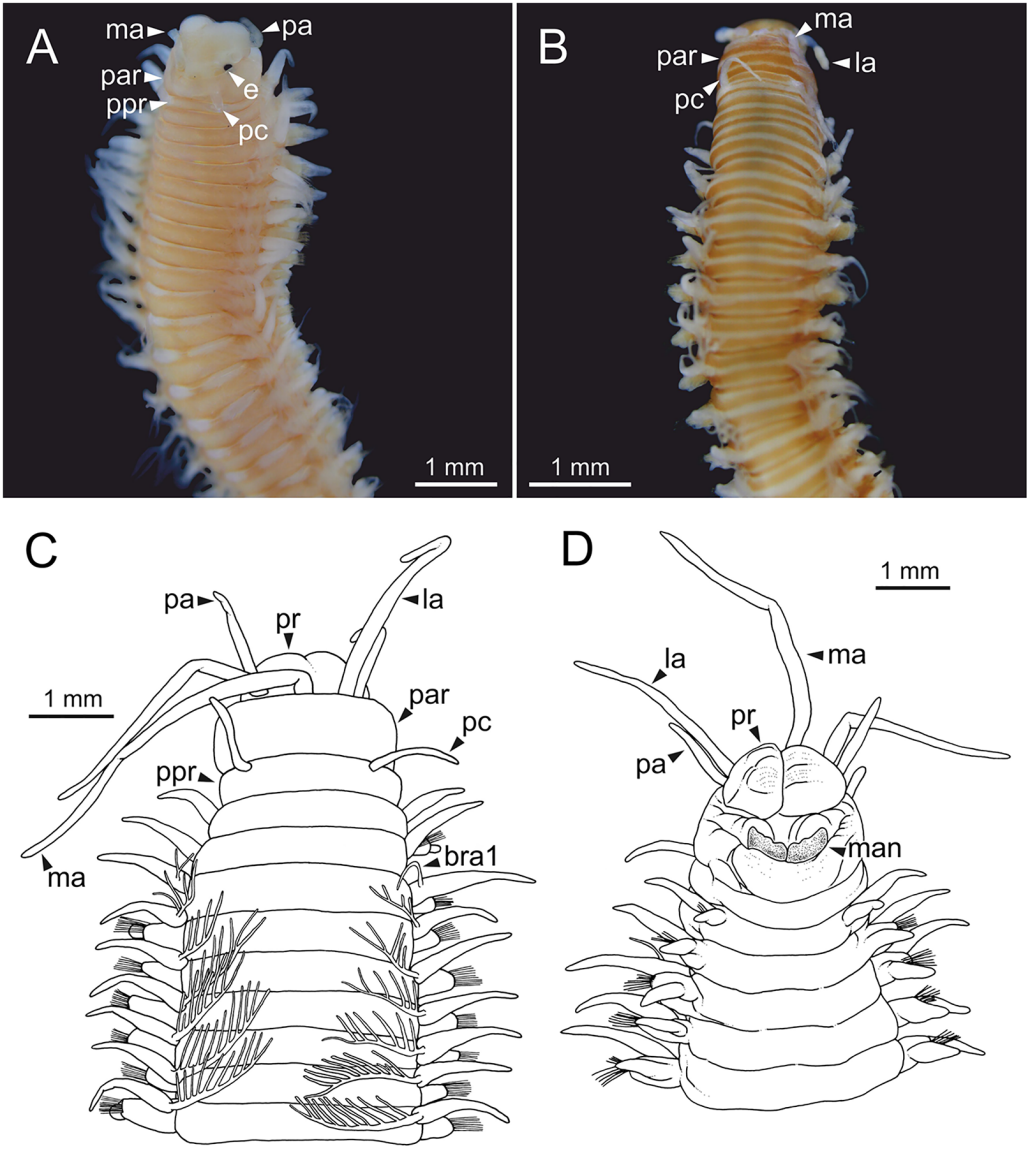
Stereomicrographs and line drawings of *Eunice woodwardi*. Three specimens (Ría de Ferrol, MNCN 16.01/19159, MNCN 16.01/19167 and MNCN 16.01/19168). (A, B) Anterior end in dorsal view of two preserved specimens. (C, D) Line drawings of anterior end of one preserved specimen in dorsal (C) and ventral view (D). bra1, branchia 1; e, eye; la, lateral antenna; ma, median antenna, man, mandibles; ooc, oocytes; pa, palp; par, peristomial anterior ring; pc, peristomial cirrus; ppr, peristomial posterior ring; pr, prostomium.

Body slightly flattened dorsoventrally; most specimens whitish, original colouration not preserved in alcohol. Original colouration was observed in three specimens (Figs. 2–3, 4A, 4B). One specimen studied alive showing colouration consisting of two broad dorsal dark red bands per segment separated by bands of much lighter tone, consecutive segments separated by a dark red thin band (Figs. 2–3, 4B); bands from peristomium to posterior body end, slightly fading in tone from posterior branchial region to posterior body half depending on specimen and preservation state. One preserved specimen showing a different colouration pattern, consisting of transverse dark bands much more faded, and bands of lighter tone wider (Fig. 4A).

**Figure 5:**
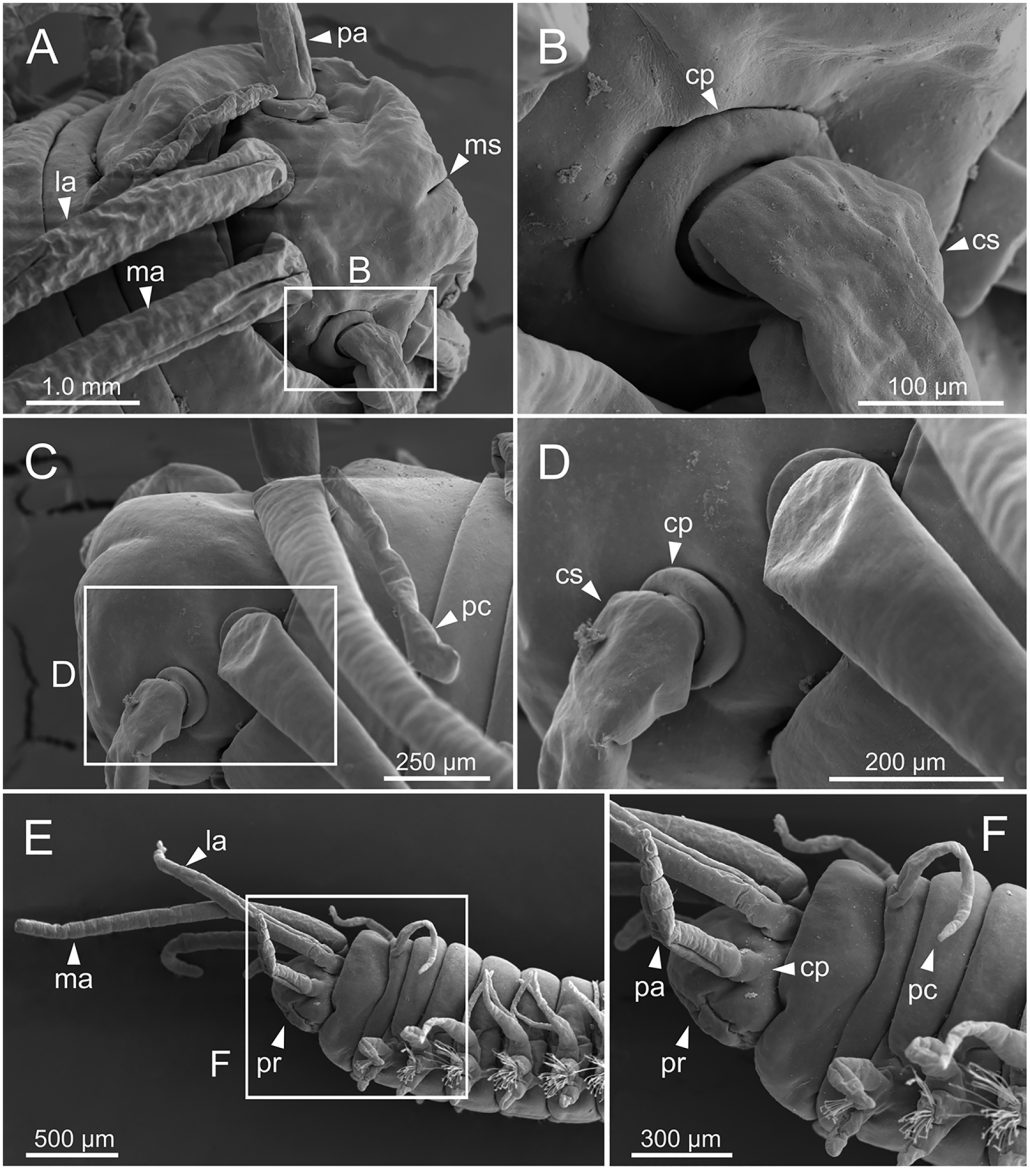
SEM micrographs of anterior end of *Eunice woodwardi*. Three specimens (Ría de Ferrol, MNCN 16.01/19144, MNCN 16.01/19155 and MNCN 16.01/19163). (A) Prostomium and peristomium, dorsal view; (B) palp base, detail of (A); (C) prostomium and peristomium, latero-dorsal view; (D) palp base and lateral antenna, detail of (C); (E) anterior end, lateral view; (F) anterior end, detail of (E). cp, ceratophore; cs, ceratostyle; la, lateral antenna; ma, median antenna; ms, median sulcus; pa, palp; pc, peristomial cirrus; pr, prostomium.

**Figure 6:**
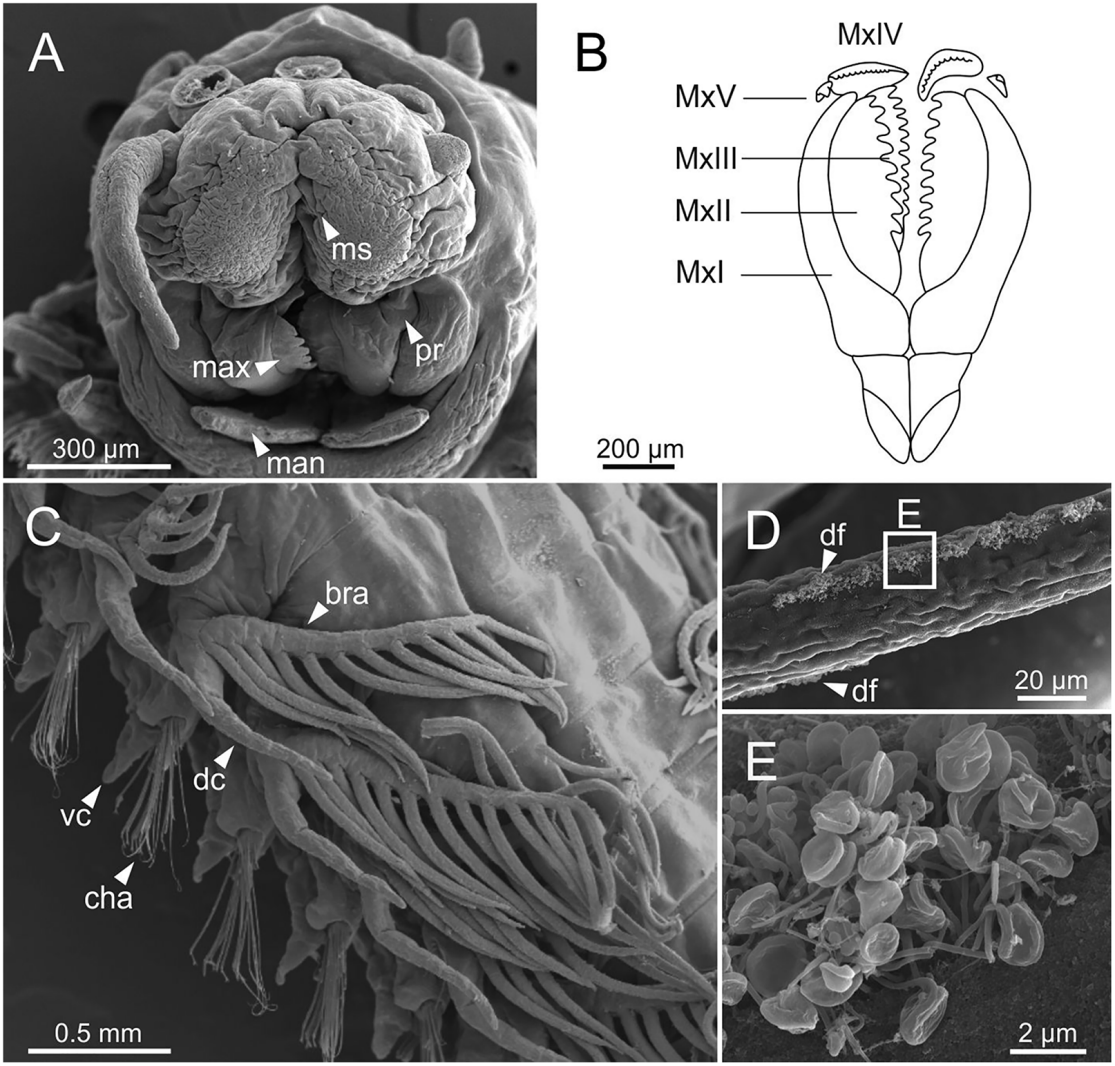
SEM micrographs of anterior end, schematic drawing of maxillae and FESEM micrographs of branchiae of *Eunice woodwardi*. Three specimens (Ría de Ferrol, MNCN 16.01/19148, MNCN 16.01/19164 and MNCN 16.01/19166). (A) Anterior end, frontal view; (B) schematic drawing of maxillae; (C) mid-body branchiae, left side, dorsal view; (D) branchial filament, detail; (E) discocilia on branchial filament, detail of (D). bra, branchia; cha, chaetae; dc, dorsal cirrus; df, fringe of discocilia; man, mandibles; max, maxillae; ms, median sulcus; MxI–V, maxilla I to V; pr, prostomium; vc, ventral cirrus.

Prostomium nearly as wide as peristomium but shorter (Figs. 2C, 2E, 4C, 5A). Prostomial lobes frontally rounded and obliquely divided (Figs. 2C, 2E, 4D). Prostomial appendages equally separated from each other, arranged in horseshoe shape; all antennae with ring-shaped ceratophores (Figs. 5B, 5D); ceratostyles slender, digitiform, smooth and lacking distinct articulations or constrictions (Figs. 2, 4C, 4D, 5A, 5C, 5E, 5F). Median antenna about 1.2 times as long as lateral antennae; palps one third as long as lateral antennae (Figs. 2A, 4C, 4D, 5E). A pair of dark eyes between lateral antennae and palps (Figs. 2A, 2B, 2D, 3A, 4A). Peristomium cylindrical; anterior ring longer than posterior one (Figs. 2B, 2D, 4C); peristomial cirri digitiform, smooth, thinner than palps (Figs. 2B, 2D, 3A, 4C). Mandibles flat (Fig. 6A). Maxillary formula: MxI: 1+1, MxII: 8+9–10, MxIII: 9–11+0, MxIV: 10–11+11, MxV: 1+1, MxVI: absent. Mx III behind left MII. Left MxIV wider than long. MxV fang-shaped (Fig. 6B).

**Figure 7:**
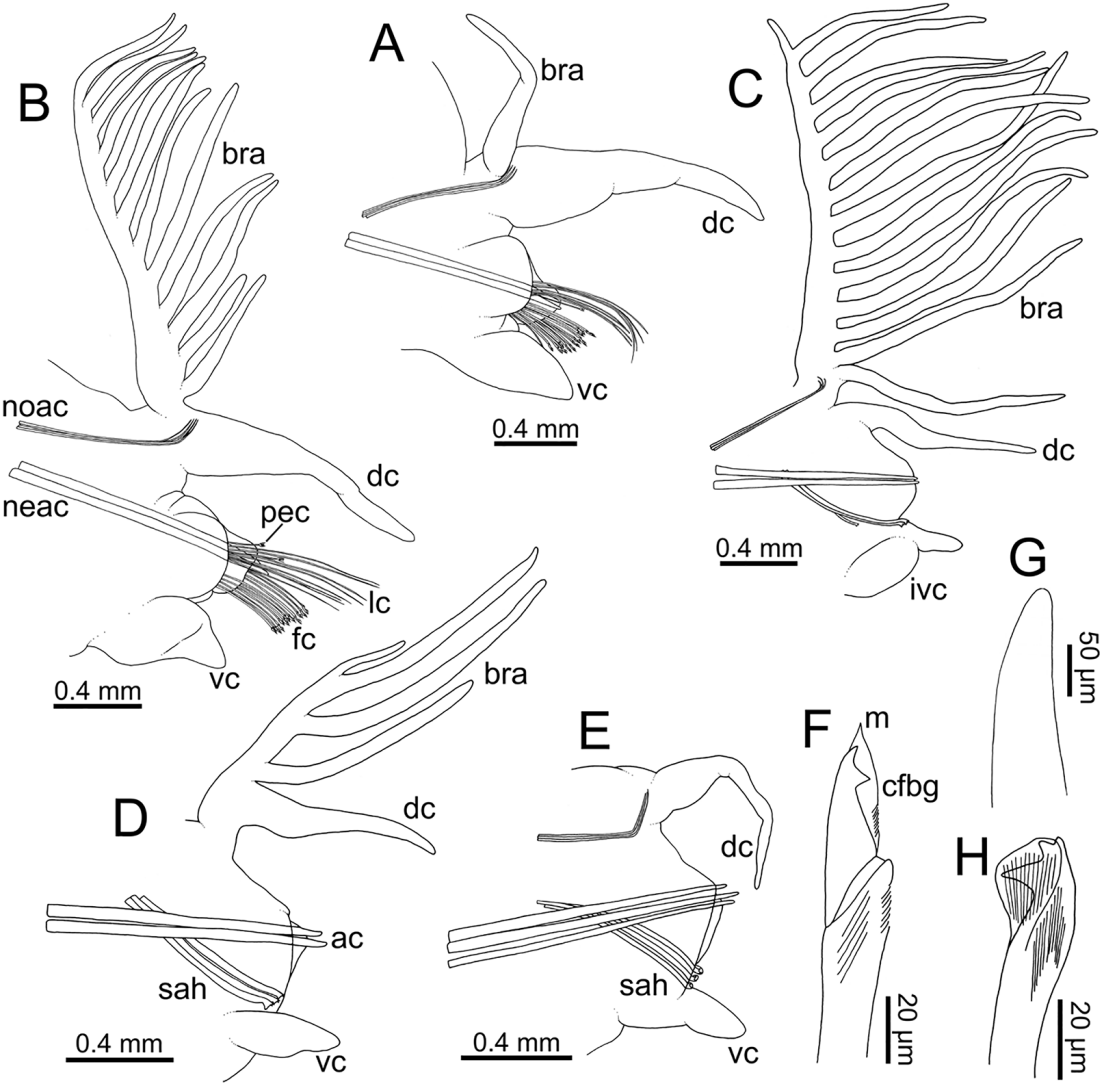
Line drawings of parapodia in posterior view and chaetae of *Eunice woodwardi*. Two specimens (Ría de Ferrol, MNCN 16.01/19152 and MNCN 16.01/19159). (A) Parapodium, chaetiger 3; (B) parapodium, chaetiger 10; (C) parapodium, chaetiger 31; (D) parapodium, chaetiger 42; (E) parapodium, chaetiger 44; (F) compound falciger chaeta; (G) acicula; (H) subacicular tridentate hook. ac, acicula; bra–branchia; cfbg, compound falciger blade guard; dc, dorsal cirrus; fc, falciger chaeta; ivc, inflated ventral cirrus; lc, limbate chaeta; m, mucro, neac, neuroaciculae; noac, notoaciculae; pec, pectinate chaeta; sah, subacicular hook; vc, ventral cirrus.

Branchiae pectinate, stem longer than dorsal cirri (Figs. 2A, 3C, 4C, 6C), from chaetiger 3 to 36–46 (<55% of body chaetigers); first branchia with 1–2 filaments (Figs. 4C, 5E, 7A); up to 9–14 filaments per branchia in following chaetigers. Chaetigers 15–25 with maximum number of filaments (Figs. 2A, 3A–3C, 6C, 7B–7D), last branchiate chaetigers with 1–2 filaments (Figs. 3A, 3D). Branchial filaments longer or about as long as dorsal cirri in mid-branchial region; filaments showing two longitudinal fringes of discocilia (Figs. 6D, 6E).

Parapodia sub-biramous. Dorsal cirri smooth, digitiform, tapering. Ventral cirri short with a digitiform tip (Figs. 7A–7E), inflated basally from about chaetiger 3 to 40; starting in chaetiger 3 in all specimens (Fig. 7C). Variation in length and width of dorsal and ventral cirri depending on specimen size, decreasing in length from anterior to posterior parapodia (Table S1). Lateral interramal red dots present in almost all body segments.

**Figure 8:**
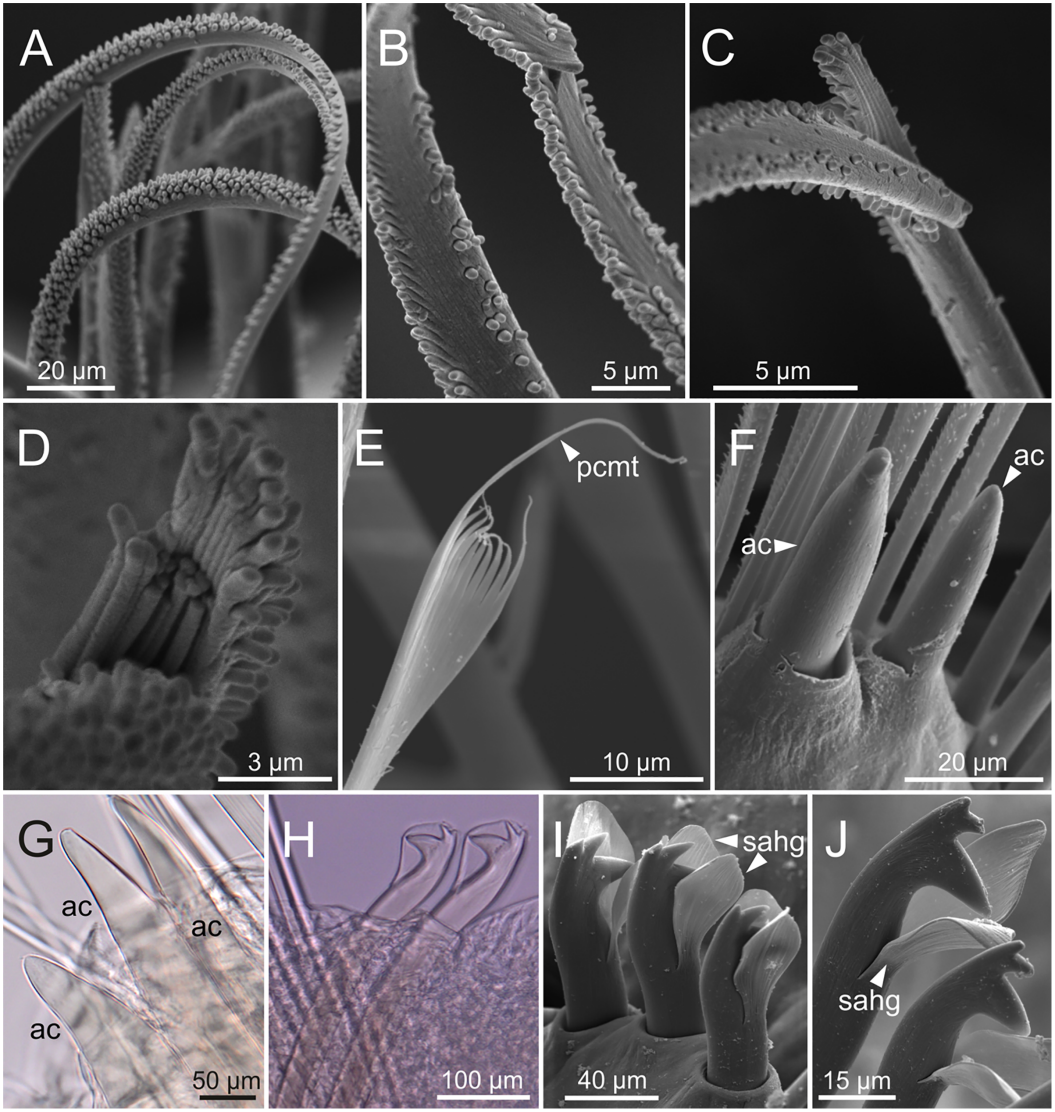
SEM and LCM micrographs of chaetae of *Eunice woodwardi*. Five specimens (Ría de Ferrol, MNCN 16.01/19150, MNCN 16.01/19153, MNCN 16.01/19155, MNCN 16.01/19156 and MNCN 16.01/19163). (A) Limbate chaetae; (B–D) detail of a subdistal breakage of one limbate chaeta; (E) pectinate chaeta; (F, G) neuroaciculae, distal end; (H–J) subacicular hooks, distal end. ac, acicula; pcmt, pectinate chaeta marginal teeth; sahg, subacicular hook guard.

**Figure 9:**
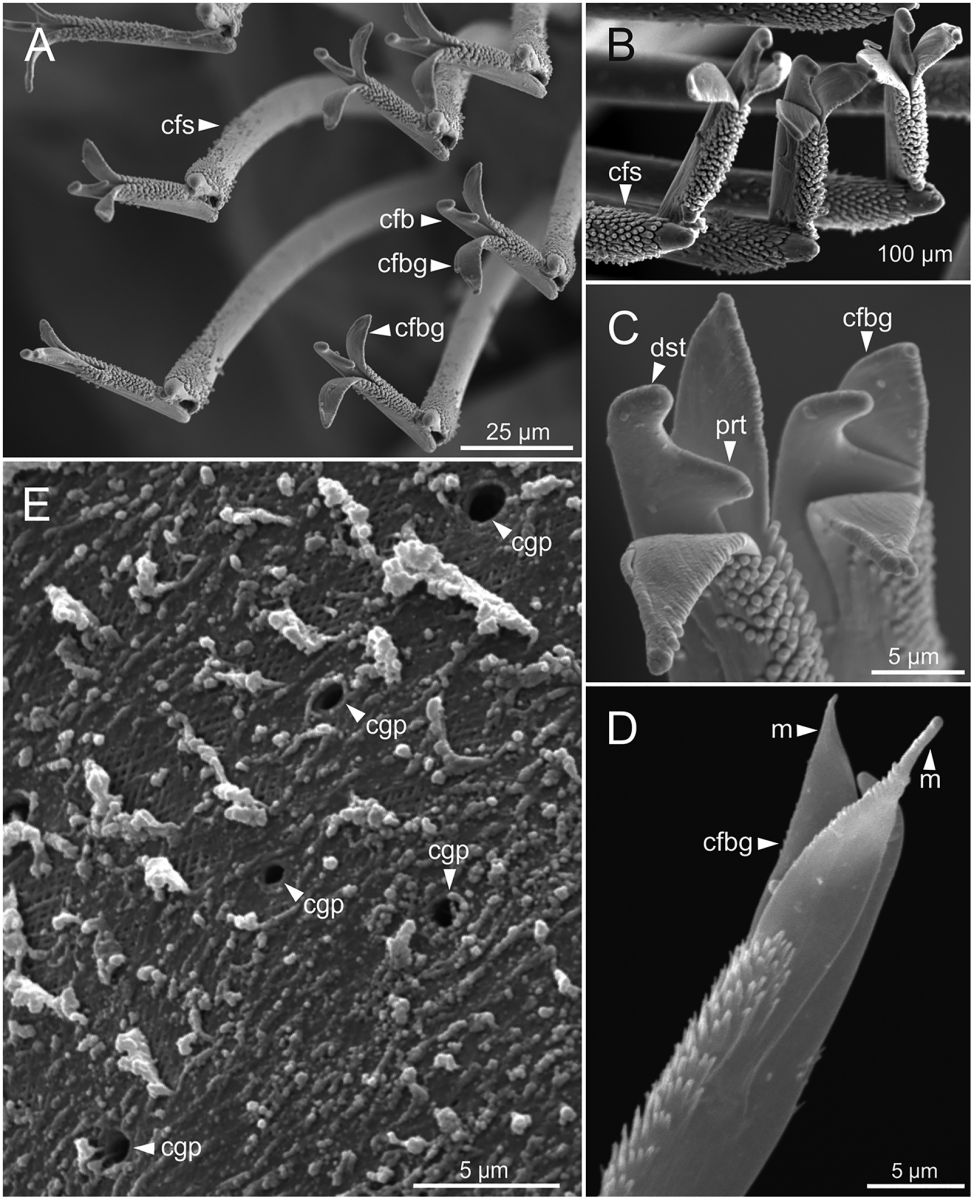
SEM micrographs of falciger chaetae and cuticular pores of *Eunice woodwardi*. Four specimens (Ría de Ferrol, MNCN 16.01/19144, MNCN 16.01/19155, MNCN 16.01/19158 and MNCN 16.01/19163). (A, B) Falciger chaetae, detail of blade and shaft distal end; (C, D) falciger chaetae, detail of blade distal end and guards; (E) cuticular glandular pores. cfb, compound falciger blade; cfbg, compound falciger blade guard; cfs, compound falciger shaft; cgp, cuticular glandular pore; dst, distal tooth; m, mucro; prt, proximal tooth.

Pre- and postchaetal lobes low, transverse folds. Prechaetal lobe fold covering proximal third of compound falcigers shaft. Acicular lobes truncate (Figs. 7A–7C). 3–4 notoaciculae, thin, distally bent (Figs. 7A–7C, 7E); two (sometimes three) yellow neuroaciculae, one larger than the other, all neuroaciculae tapering with blunt tips, curved distally and protruding from acicular lobe, never T-shaped (Figs. 7A–7E, 7G, 8F, 8G). Chaetae including 3–14 limbate, 1–4 pectinate, 3–23 compound falcigers and 1–5 tridentate subacicular hooks. Limbate and pectinate chaetae arranged in a bundle dorsal to neuroaciculae (Figs. 7A, 7B). Limbate chaetae elongated, marginally serrated (Figs. 8A–8D), distally curved and tapering (Figs. 7A, 7B). All pectinate chaetae heterodont (Fig. 8E), about 0.3–0.4 times as long as limbate chaetae; 7–9 teeth and one external tooth three times as long as others. Compound falcigers ventral to neuroaciculae (Figs. 7A, 7B); shafts distally inflated and marginally serrated (Figs. 7F, 9A, 9B). Blades bidentate (Figs. 7F, 9A–9C); proximal tooth slightly larger than distal tooth, triangular, perpendicular to blade axis; distal tooth curved dorsally (Fig. 9C); blade distal two thirds protected by elongated guard, marginally serrated (Figs. 9A–9C) and provided with conspicuous apical mucro (Figs. 7F, 9D). Number of limbate and compound falcigers decreasing from anterior to posterior chaetigers (Tables S2, S3). Subacicular hooks from chaetigers 16–31 to posterior region (Fig. 7H), ventral to neuroaciculae (Figs. 7C–7E), yellowish, tridentate with teeth in a crest, distal end protected by rounded guards (Figs. 8H–8J); size decreasing towards posterior region. Number of hooks size-depending, numbering 3 at least; increasing from 1 up to 5 from chaetiger 25–30 to middle-posterior region and then decreasing to 1–2 towards posterior region. Hooks almost hidden within parapodia in anterior chaetigers and protruding conspicuously in last chaetigers.

Pygidium with two pairs of cirri, one pair two times as long as other, smooth, non-articulated.

Cuticular glandular pores present throughout body surface (Fig. 9E).

**Internal anatomy**

**Figure 10:**
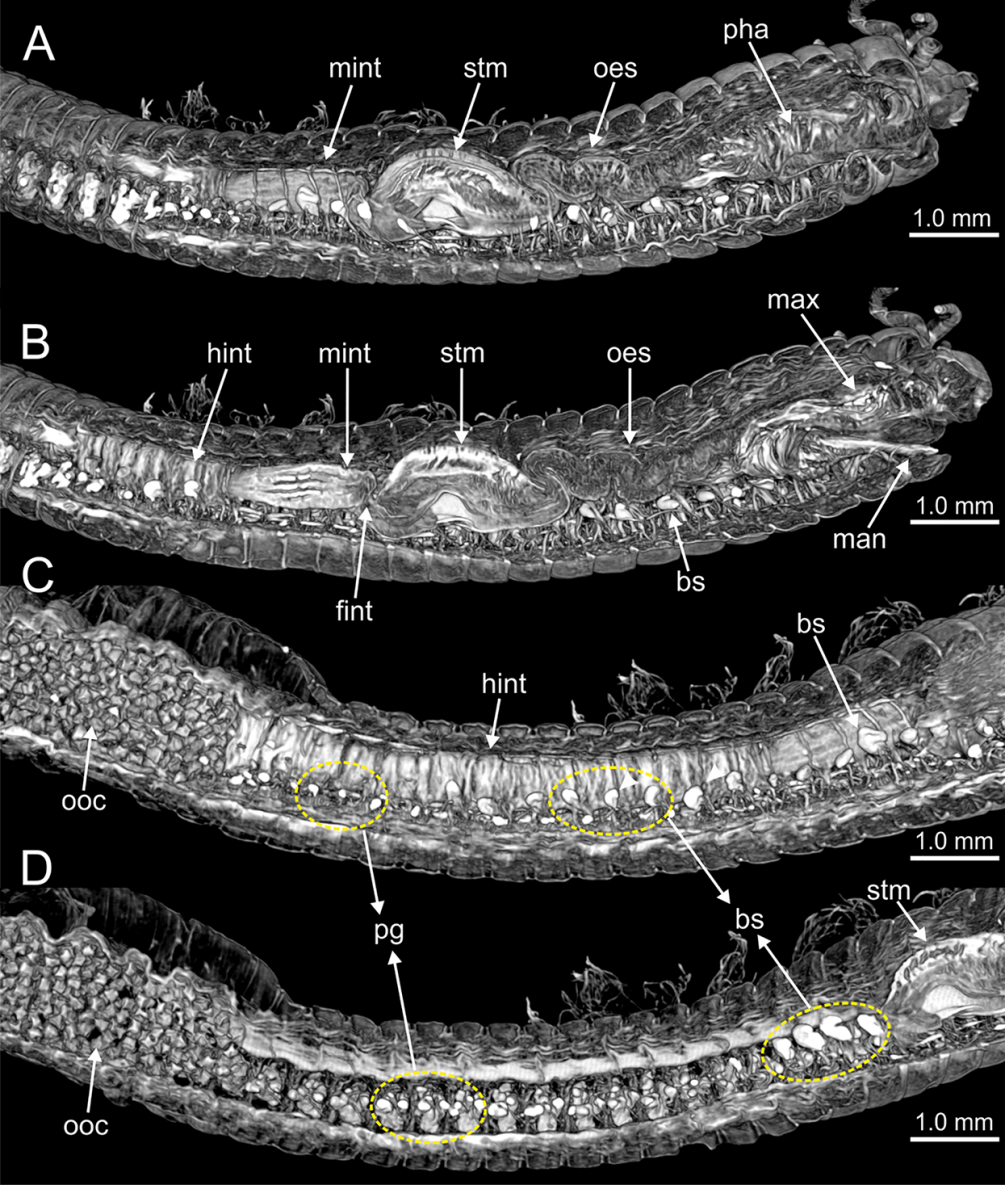
Micro-CT 3D volume renderings of *Eunice woodwardi*. Internal anatomy of one specimen (Ría de Ferrol, MNCN 16.01/19149). (A–D) Four sagittal sections showing regionalization of the digestive tract. bs, blood sinus; fint, fore intestine; hint, hind intestine; man, mandibles; max, maxillae; mint, mid-intestine; oes, oesophagus; ooc, oocytes; pg, parapodial gland; pha, pharynx; stm, stomach.

**Figure 11:**
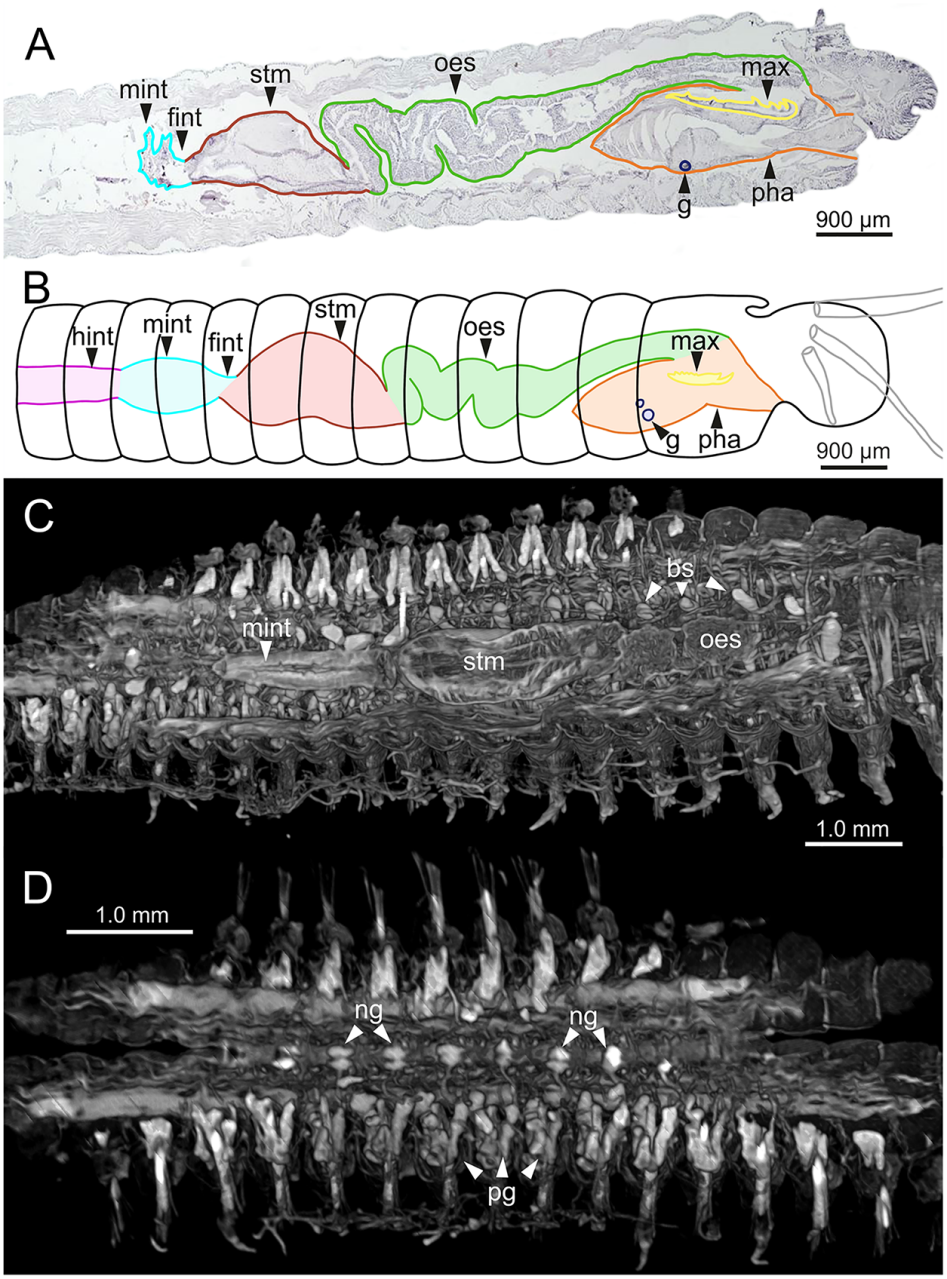
Histological sections, schematic reconstruction and micro-CT 3D volume renderings of *Eunice woodwardi*. Internal anatomy of two specimens (Ría de Ferrol, MNCN 16.01/19148 and MNCN 16.01/19149). (A) Composite image of four histological sagittal sections showing internal body organization; (B) schematic reconstruction of digestive tract; (C, D) two micro-CT frontal sections showing main internal anatomical features. bs, blood sinus; fint, fore intestine; g, gland; hint, hind intestine; max, maxillae; mint, mid-intestine; ng, nerve ganglia; oes–oesophagus; pg, parapodial gland; pha, pharynx; stm, stomach.

**Figure 12:**
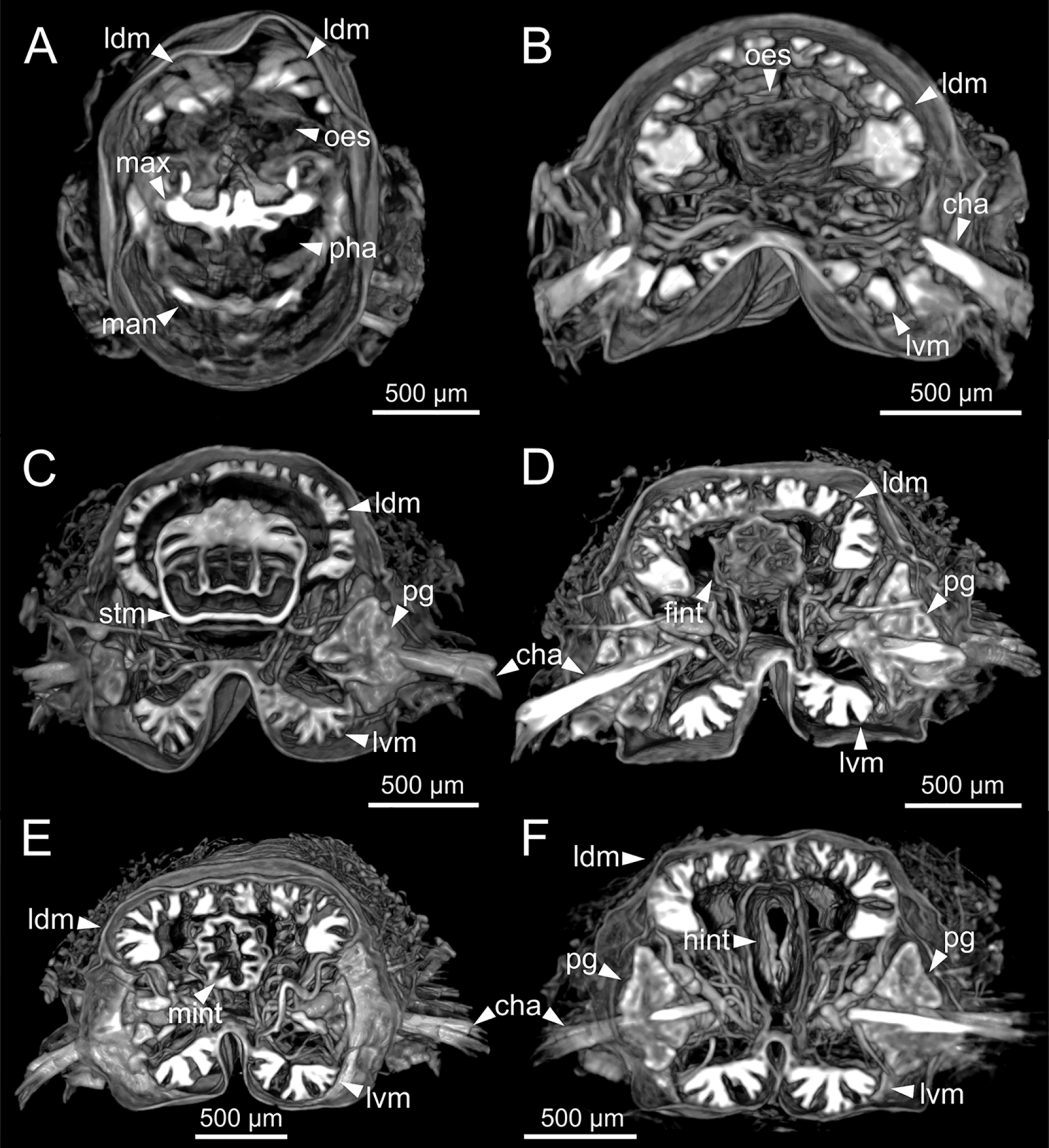
Micro-CT 3D volume renderings of *Eunice woodwardi*. Transversal sections showing internal anatomy of one specimen (Ría de Ferrol, MNCN 16.01/19143). (A) Pharynx; (B) oesophagus; (C) stomach; (D) fore intestine; (E) mid-intestine; (F) hind intestine. cha, chaetae; fint, fore intestine; hint, hind intestine; ldm, longitudinal dorsal muscles; lvm, longitudinal ventral muscles; man, mandibles; max, maxillae; mint, mid intestine; oes, oesophagus; pg, parapodial gland; pha, pharynx; stm, stomach.

**Figure 13:**
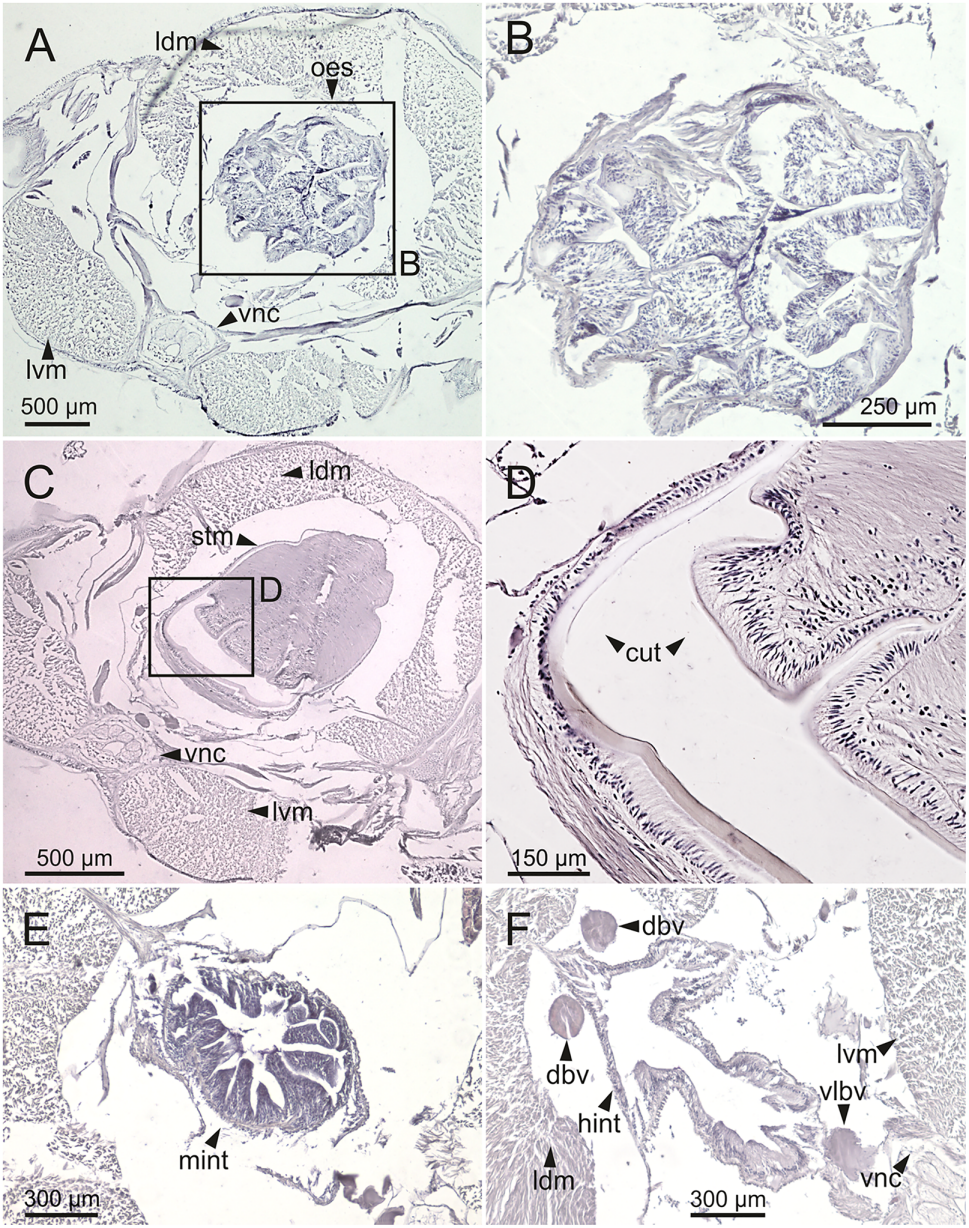
Histological sections of *Eunice woodwardi*. Transversal sections showing regions of the digestive tract of one specimen (Ría de Ferrol, MNCN 16.01/19147). (A, B) Oesophagus; (C, D) stomach; (E) mid-intestine; (F) hind intestine. cut, cuticle; dbv, dorsal blood vessel; hint, hind intestine; ldm, longitudinal dorsal muscles; lvm, longitudinal ventral muscles; mint, mid intestine; oes, oesophagus; stm, stomach; vlbv, ventral longitudinal blood vessel; vnc, ventral nerve cord.

**Figure 14:**
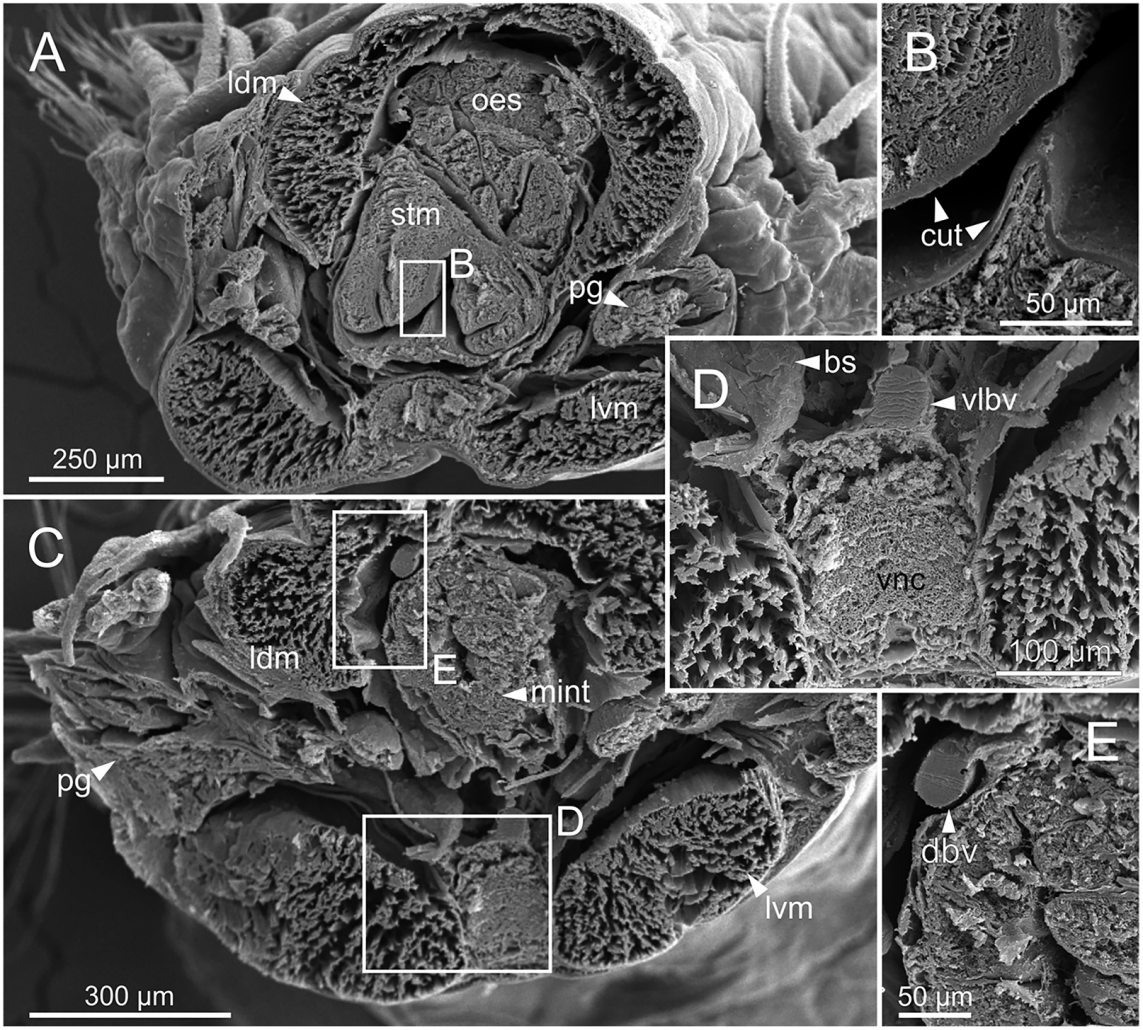
SEM micrographs of *Eunice woodwardi*. Transversal sections showing regions of the digestive tract of one specimen (Ría de Ferrol, MNCN 16.01/19164). (A) Oesophagus and stomach; (B) cuticle of stomach, detail of (A); (C) mid-intestine; (D, E) details of (C). bs, blood sinus; cut, cuticle; dbv, dorsal blood vessel; ldm, longitudinal dorsal muscles; lvm, longitudinal ventral muscles; mint, mid-intestine; oes, oesophagus; pg, parapodial gland; stm, stomach; vlbv, ventral longitudinal blood vessel; vnc, ventral nerve cord.

**Figure 15:**
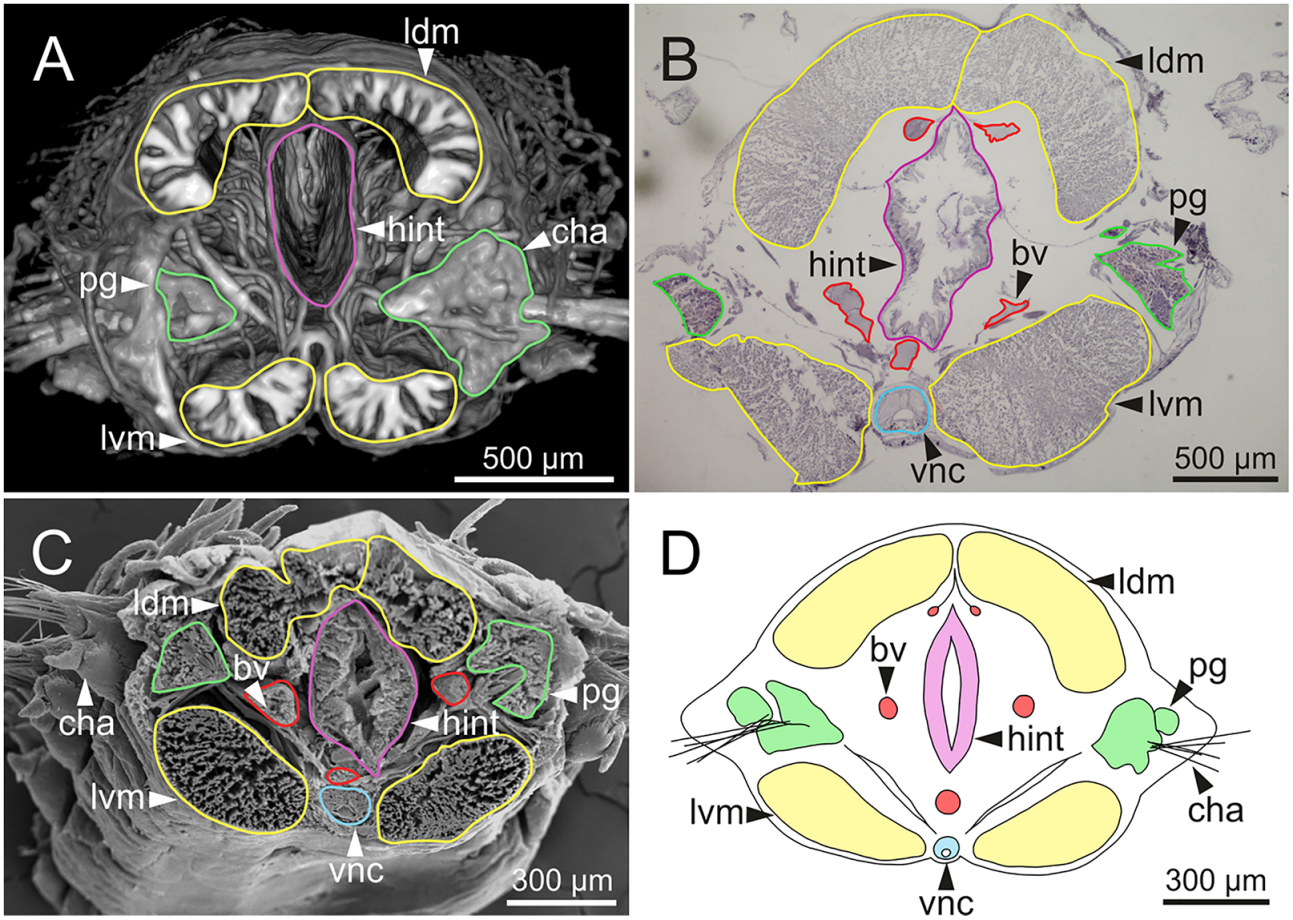
Micro-CT 3D volume rendering, histological section, SEM micrograph and schematic reconstruction of *Eunice woodwardi*. Frontal views of hind intestine of three specimens (Ría de Ferrol, MNCN 16.01/19147, MNCN 16.01/19149 and MNCN 16.01/19164). (A) Micro-CT section; (B) histological section; (C) SEM micrograph; (D) schematic reconstruction showing a transversal section depicting hind intestine (pink), longitudinal musculature (yellow), blood vessel (red), parapodial gland (green) and ventral nerve cord (blue). bv, blood vessel; cha, chaetae; ldm, longitudinal dorsal muscles; lvm, longitudinal ventral muscles; hint, hind intestine; pg, parapodial gland; vnc, ventral nerve cord.

The digestive tract is clearly regionalized and consists of: (1) pharynx (provided with mandibles and maxillae), (2) oesophagus, (3) stomach, (4) fore intestine, (5) mid-intestine, and (6) hind intestine (Figs. 10, 11A, 11B). The pharynx (Figs. 10A, 11A, 11B, 12A) is located ventrally and provided with a pair of rounded structures (g, Figs. 11A, 11B; see Discussion). The oesophagus is elongated and straight or slightly folded in shape depending on the state of pharynx protraction (Figs. 10A, 10B, 11A–11C, 12A, 12B, 13A, 13B, 14A). The oesophagus is connected to a highly muscular stomach (Figs. 10A, 10B, 10D, 11A–11C, 12C, 13C, 13D, 14A, 14B). It is followed by the fore intestine and the mid-intestine, ending in the hind intestine (Figs. 10A–10C, 11A–11C, 12D–12F, 13E, 13F, 14C, 15); the latter is a long and narrow tube provided with a very thin wall (Figs. 13F, 15).

The body musculature is well developed, particularly in the pharynx and body wall (Figs. 12, 15). It is composed by circular, dorsal and ventral longitudinal and ventral oblique muscles (Fig. 15). The longitudinal musculature is divided into four bands, two arranged dorsally and two ventrally, the latter flanking the ventral nerve cord at both sides (Figs. 13A, 13C, 14C, 14D, 15).

The circulatory system includes two longitudinal ventral and one (sometimes two) dorsal vessels (Figs. 13F, 14D, 14E). Blood masses (blood sinuses) attached to the digestive tract were also observed, being especially abundant in the oesophagus, stomach, and intestine (Figs. 10C, 10D, 11C).

A large glandular mass was observed basally in each parapodium closely associated to the chaetal bundle (Figs. 10C, 10D, 11D, 15).

**Figure 16:**
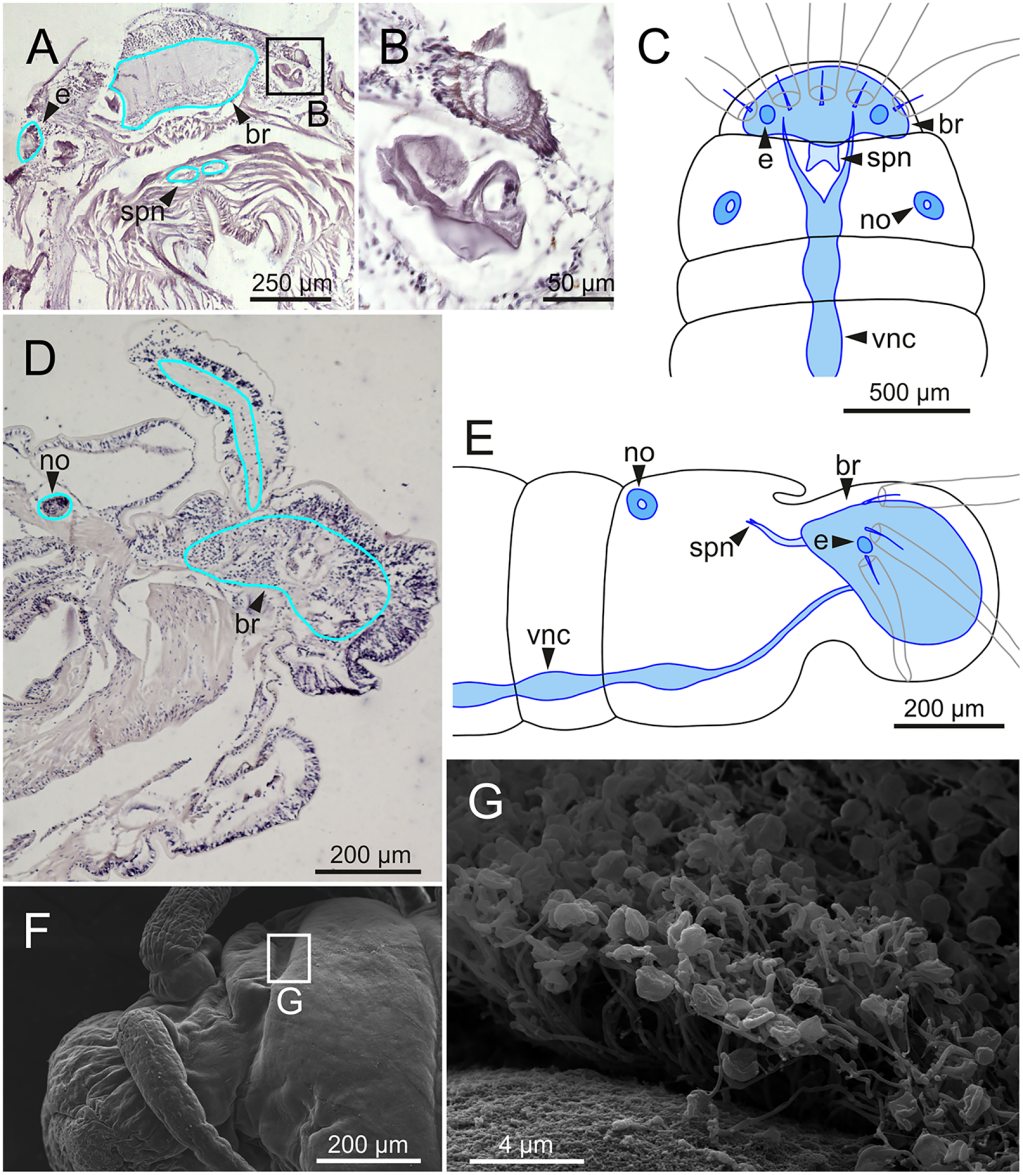
Histological section, schematic reconstructions and FESEM micrographs of *Eunice woodwardi*. Nervous system and sensory organs of three specimens (Ría de Ferrol, MNCN 16.01/19147, MNCN 16.01/19148 and MNCN 16.01/19166). (A, B) Histological frontal section showing brain and eye; (C, E) schematic reconstructions showing main nervous and sensory elements of anterior end in frontal (C) and sagittal sections (E); (D) histological sagittal section showing brain and nuchal organ; (F) SEM micrograph of prostomium and peristomium, lateral view; (G) detail of nuchal organ framed in (E). br, brain; e, eye; no, nuchal organ; spn, supra-oesophageal nerve; vnc, ventral nerve cord.

**Figure 17:**
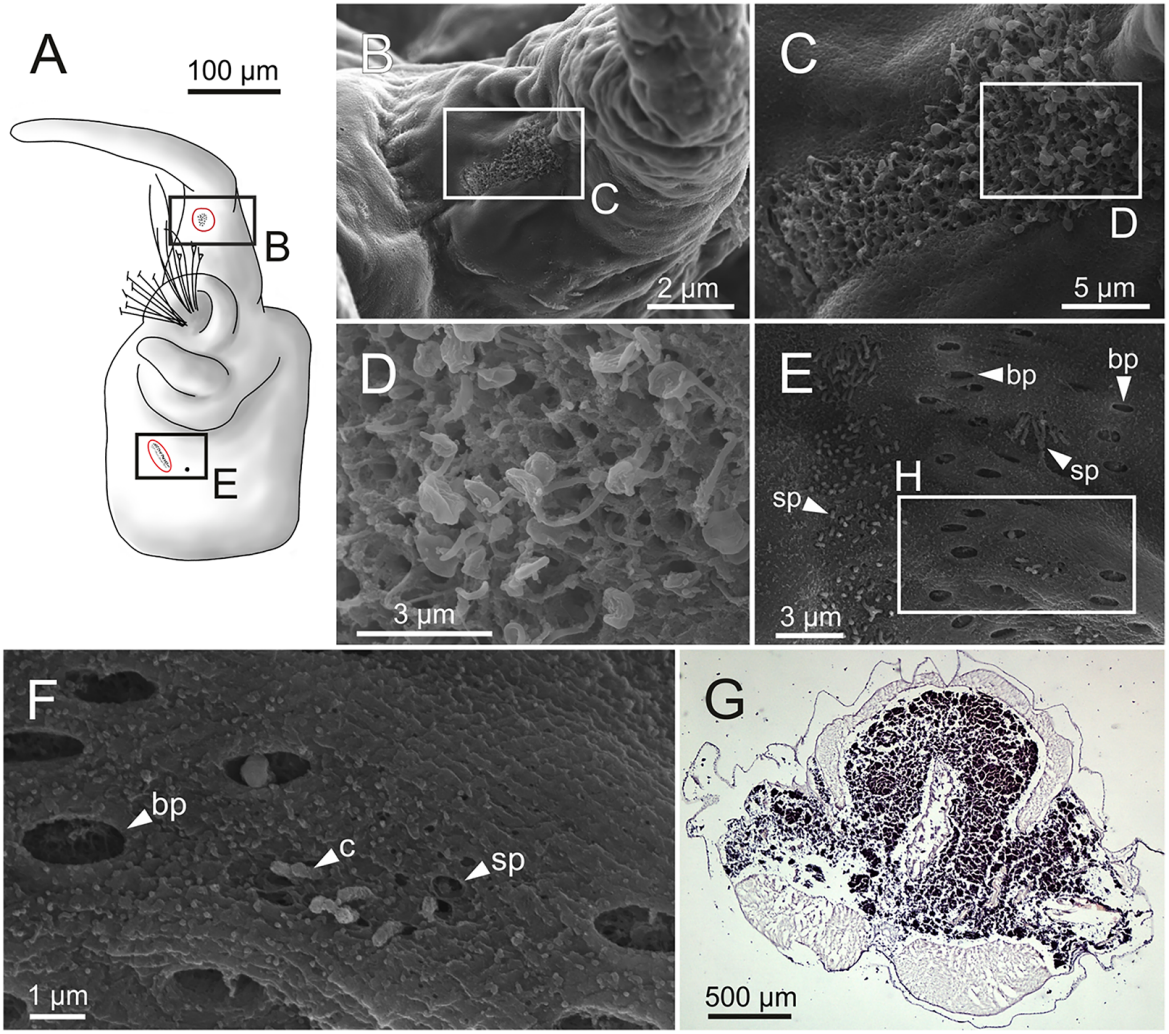
Schematic reconstruction, FESEM micrographs of parapodia and histological section of internal anatomy of *Eunice woodwardi*. Four specimens (Ría de Ferrol, MNCN 16.01/19157, MNCN 16.01/19158, MNCN 16.01/19160 and MNCN 16.01/19166). (A) Schematic reconstruction of a parapodium showing position of dorsal cirrus organ and ciliary area; (B–D) FESEM micrographs of parapodium showing dorsal cirrus organ; (C, D) details of (B); (E, F) FESEM micrographs of parapodium showing the ciliary area; (F) detail of (E); (G) histological transversal section showing body cavity full of gametes (stained in darker tones). bp, big pore; c, cilium; sp, small pore.

The central nervous system comprises a well-developed brain (Figs. 16A–16E), supra-oesophageal nerves (Figs. 16A, 16C, 16E) and the typical ventral nerve cord, with pair of ganglia in same segment almost fused to each other. Several sensory organs were observed: (1) a pair of eyes located slightly behind lateral antennae (Figs. 16A, 16C, 16E), (2) a pair of nuchal organs in posterior half of anterior peristomium ring (Figs. 16C–16G), and (3) ciliary areas located in proximal third of dorsal cirri in all chaetigers (Figs. 17A–17D); the aforementioned cilia correspond to discocilia (Figs. 17C, 17D).

Another ciliary area of unknown function was observed ventrally to ventral parapodial cirrus (Figs. 17A, 17E). The epidermis nearby this area in mid-body segments has several pores arranged in two groups next to each other; the larger pores are about 1 µm in diameter and the smaller ones 0.2–0.3 µm. Each pore is provided with a cilium (Figs. 17E, 17F).

**Figure 18:**
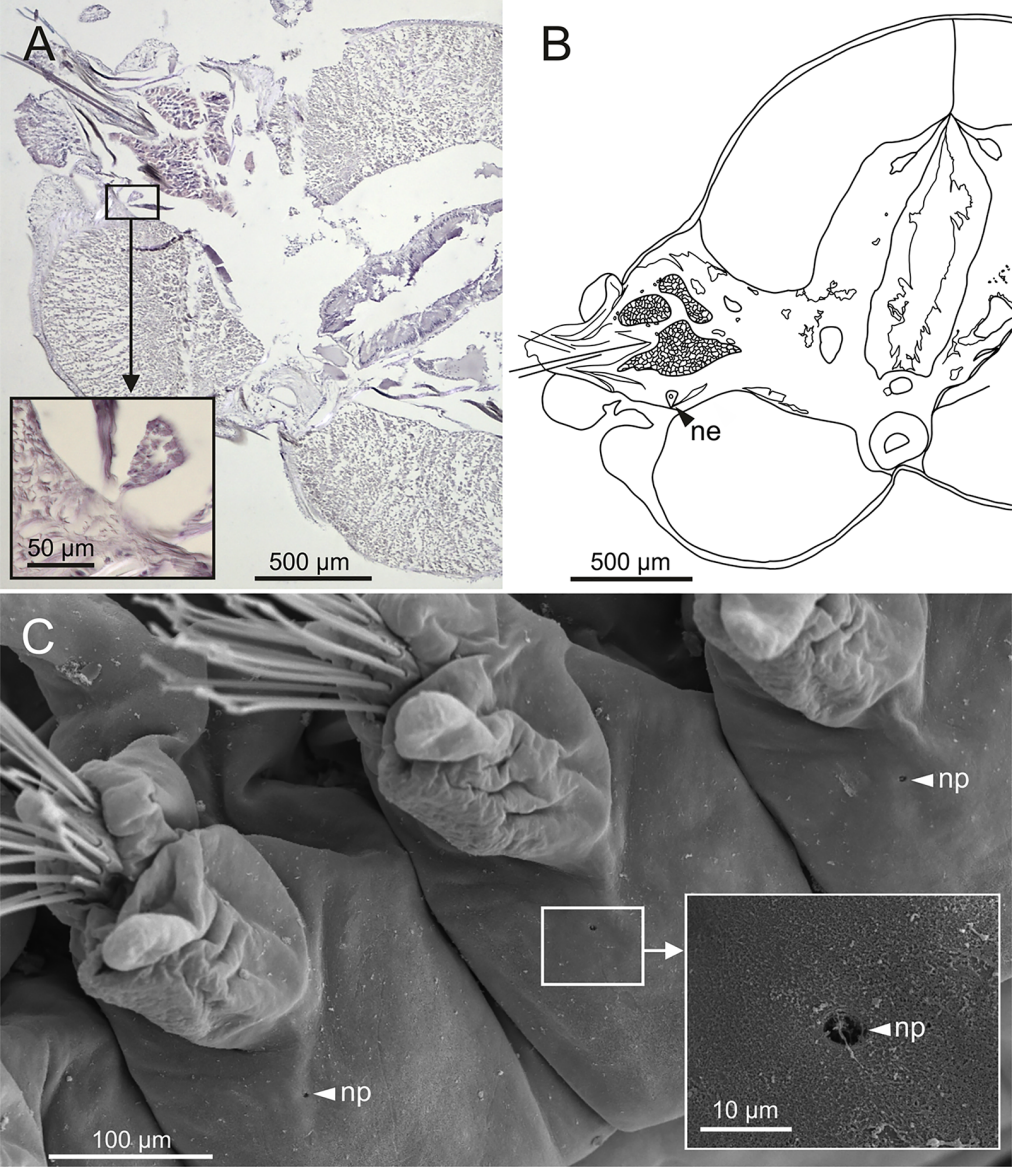
Histological section, schematic reconstruction and SEM micrographs of nephridia of *Eunice woodwardi*. Two specimens (Ría de Ferrol, MNCN 16.01/19144 and MNCN 16.01/19147). (A) Histological transversal section showing position of a nephridium (framed); (B) schematic reconstruction of (A); (C) SEM micrograph showing position of three nephridiopores, latero-ventral view, and detail of nephridial pore (frame). ne, nephridium; np, nephridial pore.

Nephridia appear as glandular masses arranged metamerically that were observed in most of body segments apart from the anteriormost and posteriormost segments (Fig. 18). These are located ventro-laterally between the ventral longitudinal musculature and the glandular masses associated with chaetal bundles, measuring 70–80 µm (Figs. 18A, 18B) and directly connected with nephridiopores, each 3–4 µm in diameter (Fig. 18C).

The presence of gametes in coelomic body cavity was observed with LCM, micro-CT and HIS (Figs. 1A, 10C, 10D, 17G); specimens observed with micro-CT and HIS were collected in September-November. The specimen subjected to HIS bears gametes at an early stage of maturation and corresponds probably to a male due to the small gamete size (about 4–5 µm in diameter), high numbers and grouping shape (Fig. 17G). In anterior body half, gametes seem to be associated to the parapodial area, whereas in posterior half, gametes fill all the coelomic cavity. Muscular bands are less developed in posterior body half, where gametes are especially abundant; this region also lacks glandular masses associated with chaetae, blood vessels and nephridia (Fig. 17G). Specimens observed with LCM and micro-CT (Figs. 1A, 10C, 10D) are females with oocytes in an advanced stage of maturation due to their large size (150–200 µm). It was not possible to identify the location of gonads in the studied specimens; >50% of specimens were incomplete, with the posterior end missing.

**Epibiosis**

**Figure 19:**
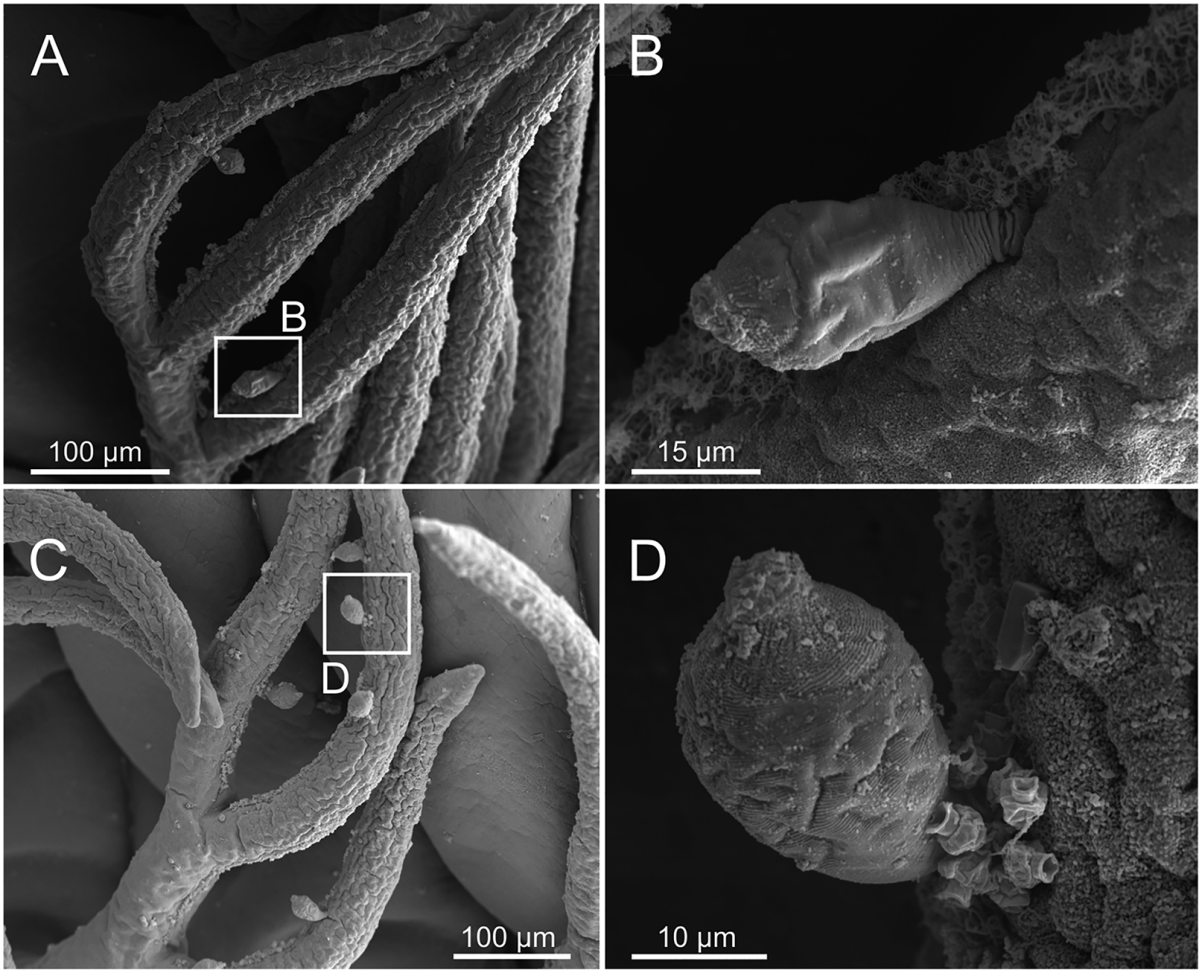
SEM micrographs of ciliophoran epibionts on *Eunice woodwardi*. One specimen (Ría de Ferrol, MNCN 16.01/19156). (A, C) Branchial filaments showing location of ciliophoran epibionts (B, D) detail of ciliophoran epibionts as shown in (A) and (C), respectively.

Ciliophorans were observed attached to the surface of branchial filaments (Fig. 19) but not showing any defined pattern of attachment or distribution.

**Distribution and ecology**

The type locality corresponds to Ría de A Coruña (Galicia, NW Spain). Unfortunately, Baird (1869) did not provide neither the coordinates nor depth or abiotic characteristics where this specimen was collected. Non-type specimens collected in the Ría de Ferrol were found from the intertidal to 26.5 m depth, in a wide range of bottom types, from coarse (gravel) to fine (sandy mud and mud) sediments (Table 1).

***Eunice vittata* (Delle Chiaje, 1829)**

*Nereis vittata*Delle Chiaje, 1829: 195.

*Eunice vittata* (Delle Chiaje). Grube, 1850: 293; Fauchald, 1992: 337–339, Fig. 115a-i, Tables 18, 41–42.

**Material examined**

Non-type material (Table 1): 2 specimens (MNHW; Naples, Italy), 5 specimens (ZMH-V 12932; Venice, Italy), 2 specimens (ZMH-P 14276, Banyuls-sur-Mer, France), 5 specimens (MNCN 16.01/2677 and 16.01/2723; Valencia, Spain) and 4 specimens (SPR04-03, PAD04-03, CBR01-06 and CBR01-11, Mallorca, Spain).

**External morphology (specimens from Naples)**

Two specimens; one complete: 24 mm long, 2 mm wide, with 84 chaetigers, and one incomplete: 23 mm long, 2 mm wide, with 80 chaetigers.

**Figure 20:**
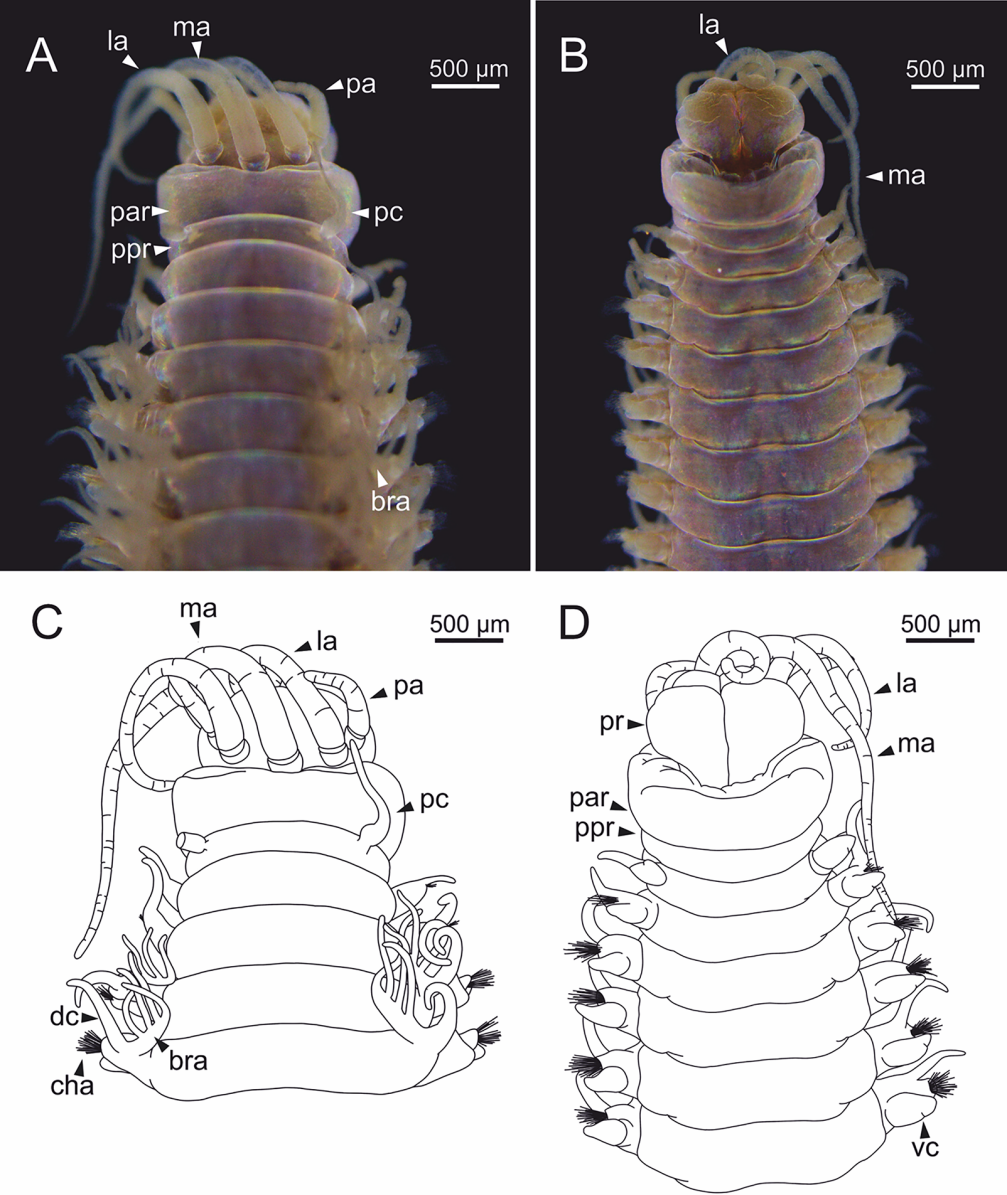
Stereomicrographs and line drawings of *Eunice vittata*. One specimen (Naples, MNHW). (A, B) Anterior end in dorsal view and ventral view. (C, D) Line drawings of anterior end in dorsal (C) and ventral view (D). bra, branchia; cha, chaetae; dc, dorsal cirrus; la, lateral antenna; ma, median antenna; pa, palp; par, peristomial anterior ring; pc, peristomial cirrus; ppr, peristomial posterior ring; pr, prostomium; vc, ventral cirrus.

Body slightly flattened dorsoventrally; original colouration not preserved in alcohol (Figs. 20A, 20B).

Prostomium nearly as wide as peristomium but shorter. Prostomial lobes frontally rounded. Prostomial appendages equally separated from each other, arranged in horseshoe shape; all antennae with ring-shaped ceratophores; ceratostyles slender, digitiform, smooth, lacking articulations but with poorly marked constrictions (Figs. 20C, 20D). A pair of dark eyes between lateral antennae and palps. Peristomium cylindrical; peristomial cirri digitiform, smooth.

Branchiae pectinate, stem longer than dorsal cirri, present from chaetiger 3 to 27–29 (<55% of body chaetigers); first branchia with 3 filaments (Fig. 20C); up to 7 filaments per branchia in following chaetigers. Last branchiate chaetigers with one filament. Branchial filaments longer or about as long as dorsal cirri in mid-branchial region chaetigers.

Parapodia sub-biramous. Dorsal cirri smooth, digitiform, tapering. Ventral cirri short with a digitiform tip, inflated basally from about chaetiger 3 to 27–29, in all specimens starting in chaetiger 3. Dorsal and ventral cirri decreasing in length from anterior to posterior parapodia (Figs. 20C, 20D). Lateral interramal red dots present in almost all body segments.

Two yellow neuroaciculae, one larger than the other; all neuroaciculae tapering with blunt tips, curved distally and protruding from acicular lobe, never T-shaped. Chaetae including 2–11 limbate, 1–3 pectinate, 3–23 compound falcigers and 1–5 tridentate subacicular hooks. Limbate and pectinate chaetae arranged in a bundle dorsal to neuroaciculae. Limbate chaetae elongated, marginally serrated, distally curved and tapering. All pectinate chaetae heterodont, about 0.3–0.4 times as long as limbate chaetae; number of teeth not observed, one external tooth three times as long as others. Compound falcigers ventral to neuroaciculae; shafts distally inflated and marginally serrated. Blades bidentate; proximal tooth slightly larger than distal tooth, triangular, perpendicular to blade axis; distal tooth curved dorsally; blade distal two thirds protected by elongated guard, marginally serrated; mucro absent. Number of limbate and compound falcigers decreasing from anterior to posterior chaetigers (Tables S2, S3). Subacicular hooks from chaetigers 21–22 to posterior region, ventral to neuroaciculae, yellowish, tridentate with teeth in a crest, distal end protected by rounded guards; size decreasing towards posterior region. Maximum number of hooks in posteriormost chaetigers. Hooks almost hidden within parapodia in anterior chaetigers and protruding conspicuously in last chaetigers.

## Discussion

### Taxonomy, distribution and external morphology

The study of the holotype of *E. woodwardi* and recently collected material in the NW Atlantic coast of Spain supports the validity of this species; *E. woodwardi* clearly differs from *E. vittata* mostly regarding the presence of an apical mucro in the guard of falciger chaetae blades and the number of teeth in pectinate chaetae. Furthermore, several features not previously described in *E. woodwardi* by Fauchald (1992) are first mentioned here.

As stated before, it has not been dilucidated yet the systematic position at the genus level of all species formerly included in *Eunice *sensu* lato*. Anyway, *E. woodwardi* would fit into group C-1 *sensu*Fauchald (1970) mostly because of having tridentate subacicular hooks and first branchiae present before chaetiger 10 and ending before chaetiger 100. According to the descriptions by Fauchald (1992) and Zanol, Fauchald & Paiva (2007), the closest species to *E. woodwardi* are *E. vittata* (placed in *Leodice* according to Zanol et al., 2021), *E. indica*Kinberg, 1865 and *E. unifrons* (Verrill, 1900), because of sharing the same chaetal types and having also digitiform cephalic appendages separated from each other by the same distance.

Eyes were not reported in all *Eunice* species; this character was not included in the description of *E. woodwardi* holotype neither by Baird (1869) nor by Fauchald (1992). However, the presence of a pair of eyes in specimens from the Ría de Ferrol is here confirmed. Therefore, eyes might have been present in the holotype and then eyes pigment would have faded after being fixed for preservation. The holotype was in ethanol for 153 years while non-type material from Ferrol was for 35 years.

The maxillary formula is also described here for the first time for *E. woodwardi*. Several features are shared with most species of *Eunice *sensu* lato*, such as: (1) only the left MxIII is present, (2) MxI and V bear only one tooth, and (3) lack of MxVI. However, *E. woodwardi* differs from other species in the number of teeth of MxII, MxIII and MxIV. The most similar maxillary formula is that of *E. vittata*; Fauchald (1992) describes it from specimens collected near the type locality, *i.e.*, MxI: 1+1, MxII: 9–10+9–10, MxIII: 8–9+0, MxIV: 6+8–12, MxV: 1+1. Other authors, after examining specimens of supposedly the same species from the Atlantic and Pacific (Fauvel, 1923) and Indian oceans (Day, 1967), indicated slight differences, *i.e.*, there were more teeth in MxIV (10+13) while Şahin & Çinar (2009) reported 9+10 in MxIV for specimens from the eastern Mediterranean Sea. Thus, *E. vittata* would differ from *E. woodwardi* in having more teeth in left MxII and left MxIV, and fewer in left MxIII. *Eunice indica*Kinberg, 1865 also has a similar formula, *i.e.*, MxI: 1+1, MxII: 9–11+8-11, MxIII: 8–11+0, MxIV: 7–10+13, MxV: 1+1 (Day, 1967), but this species bears more teeth in left MxII and fewer in right MxIV when compared to *E. woodwardi*; however, these specimens reported by Day (1967) from South Africa might correspond to a different species from *E. kinbergi*.

The morphology and arrangement of branchiae fit well with those described for species of group C-1, but differ in the range of branchiate chaetigers. In *E. woodwardi*, branchiae are present from chaetiger 3 to 36–46; however, a wider range of branchiate chaetigers has been reported for *E. vittata*, *e.g.*, 3–23 in specimens from Italy (Fauchald, 1992), 3 to 40–50 from Atlantic and Pacific oceans (Fauvel, 1923), 3–45 from Indian Ocean (Day, 1967) and 3–25 from Turkey (Şahin & Çinar, 2009). In the case of *E. indica*, branchiae seem restricted to fewer chaetigers (3 to 21; Day, 1967). The maximum number of branchial filaments is up to 12–14 in *E. woodwardi*, while in *E. vittata* this number varies slightly: up to 12 (Fauchald, 1992), 14 (Fauvel, 1923) and 10–20 (*sensu*Day, 1967, see comment above); *E. indica* bears from up to 8 (Fauchald, 1992) to 10–15 (Day, 1967).

On the other hand, the presence of discocilia on branchiae had previously been reported by Heimler (1978) for *Lanice conchilega* (Pallas, 1766). However, Ehlers & Ehlers (1978) stated that this type of cilia represent an artificial structure; this assumption was later confirmed by Short & Tamm (1991) who stated that these artefacts were caused probably by fixation and osmotic stress and by Göbbeler & Klussmann-Kolb (2006).

Overall features of chaetal composition (limbate, pectinate and compound falciger chaetae, tridentate subacicular hooks and aciculae) in *E. woodwardi* are also shared with several group C-1 species. However, the presence of a mucro in compound falcigers is not shared with all species of this group such as *E. unifrons* and *E. vittata*. Fauvel (1923), Day (1967) and Campoy (1982) described compound falcigers provided with long pointed guards in specimens of what they regarded as *E. vittata* from the Atlantic and Pacific oceans, Indian Ocean and Mediterranean Sea, respectively. On the contrary, Fauchald (1992) examined specimens of *E. vittata* collected near its type locality and stated that blade guards lack mucros. Furthermore, number of chaetae per parapodium also seem to differ between *E. vittata* and *E. woodwardi*. Specimens from the eastern Mediterranean identified as *E. vittata* by Şahin & Çinar (2009) bear 2–5 limbate and 2–6 falciger chaetae per parapodium instead of 3–14 and 3–23 respectively as found in *E. woodwardi*. On the other hand, the number of pectinate chaetae per parapodium is similar in both species but they differ in number of teeth: *E. vittata* has up to five *sensu*Fauchald (1992) and *E. woodwardi* bears 7–9. Numbers of subacicular hooks also vary across group C-1, numbering three or more in *E. woodwardi*, *E. indica* and *E. vittata* and 1–2 in remaining species.

In this context, it seems that features of specimens attributed to *E. vittata* from across the world show much variation. Therefore, we also examined specimens from several western Mediterranean locations that were identified as *E. vittata* all having tridentate subacicular and blade guards lacking a distinct mucro (following Fauchald, 1992). On the one hand, the specimens of *E. vittata* that were collected near the type locality (Naples, Italy) also differ from *E. woodwardi* in having: (1) cephalic appendages with constrictions; (2) branchiae limited to fewer chaetigers (chaetigers 3–29 *vs* 3–40); (3) first branchia provided with 3 filaments; (4) fewer branchial filaments (up to 7 *vs* 9–14); and (5) tridentate subacicular hooks first present from chaetigers 21–22. On the other hand, specimens of *E. vittata* from other locations show differences with both *E. vittata* from Naples and *E. woodwardi*. For instance, specimens from Banyuls-sur-Mer and Mallorca bear cephalic appendages that are provided with constrictions but they differ in branchial distribution range; specimens from Valencia bear branchiae from chaetiger 3 to 36–40, while those from Banyuls-sur-Mer bear fewer branchiate chaetigers (chaetiger 3 to 26–29 and 3 to 28, respectively). They also differ in maximum number of branchial filaments: 2–3 (Banyuls-sur-Mer), 4 (Mallorca) and 7 (Valencia). The number of limbate and compound falciger chaetae per parapodium also shows variation (Tables S2, S3): 3–9 limbate and 2–13 compound falcigers (Valencia) and 3–7 and 4–13 (Mallorca), that are fewer than those found in *E. woodwardi* (3–14 and 3–23). Regarding subacicular hooks, *E. vittata* from Venice showed up to four hooks per chaetiger but never reaching up to five, while *E. vittata* from Valencia were characterised by having just up to two hooks but only present in the last chaetigers (107–110) of large specimens. In all, these observations support that *E. woodwardi* from NW Iberian Peninsula differ clearly from specimens attributed to *E. vittata* from western Mediterranean; the morphological variability found among the latter also suggests that there might be several species involved that share non-mucronate blade guards and tridentate hooks, not discarding the presence of exotic species as well (Zanol et al., 2021). In this context, Zanol et al. (2021) also states that *E. vittata* (as *Leodice*) and other species formerly considered as cosmopolitan might have restricted distributions once their taxonomy is clarified.

Furthermore, the material examined from the Ría de Ferrol corresponds entirely to *E. woodwardi*. Therefore, we also suggest that many (if not all) previous reports of *E. vittata* from NW Iberian Peninsula might correspond to *E. woodwardi*. In fact, *E. vittata* has never been reported in the Ría de Coruña, neither in the intertidal band (García Álvarez et al., 1993) nor in the subtidal realm (López-Jamar & Mejuto, 1985, 1988; López-Jamar, González & Mejuto, 1986; López-Jamar & González, 1987; López-Jamar et al., 1995).

### Internal anatomy

The study of several specimens with the micro-CT and through HIS has revealed for the first time the main features of the internal anatomy of *E. woodwardi*, such as the regionalized digestive tract or the presence of nuchal organs. The highly regionalized gut of *E. woodwardi*, divided into pharynx, oesophagus, stomach, fore, mid- and hind intestine, is against the opinion of Penry & Jumars (1990), *i.e*., that the digestive tract in carnivorous “polychaetes” is very simple, and divided only into two parts, a foregut and a hindgut. However, these authors did not include in their study neither carnivorous taxa nor any *Eunice* species. Anyway, we have not been able to verify whether *E. woodwardi* actually behave as a carnivore or not, and available information for other species is contradictory. For instance, Gaston (1987) considered *E. vittata* as a detritivore after examination of gut contents while Deudero et al. (2011) regarded it as a carnivore after analysing stable isotopes of carbon and nitrogen. In fact, the concept of a simple digestive tube in carnivorous species, made up of mouth, pharynx, oesophagus and intestine, had been already described by Ehlers (1868) for *Eunice*; however, the illustrations included in his work suggest that the digestive tract is more complex. The pharynx of *E. woodwardi* is ventral and may correspond to the Type 4 described by Tzetlin & Purschke (2005). We also observed a pair of small, rounded structures inside the pharynx (g, Figs. 11A, 11B), that could be similar to the glands mentioned by these authors in the same type of pharynx present in the family Dorvilleidae Chamberlin, 1919.

The arrangement of musculature, circulatory and nervous systems in *E. woodwardi* agrees with the typical pattern described for the annelid body plan (Beesley, Ross & Glasby, 2000; Tzetlin & Purschke, 2005). In fact, the central nervous system of *E. woodwardi*, composed by pairs of ganglia, almost totally fused, and located along the ventral nerve cord, is similar to that described by Ehlers (1868) for *Leodice harassii* (Audouin & Milne Edwards, 1833), Hofmann (1974) for *Palola siciliensis* (Grube, 1840) and Zanol (2010) for Eunicidae. Furthermore, different types of sensory organs such as eyes, nuchal organs and dorsal cirrus ciliary organs have been observed in *E. woodwardi*. Shape and location of nuchal organs are the same as described by Hofmann (1974) for *P. siciliensis* and Fauchald & Rouse (1997) for other members of Eunicidae. Similarly, features of dorsal cirrus organs agree with that of *Leodice antennata* Savigny *in* Lamarck, 1818 and *Marphysa sanguinea* (Montagu, 1813) (see Hayashi & Yamane, 1994). Ciliary areas observed below the parapodial ventral cirrus, of unknown function, might either correspond to the opening of parapodial glandular organs (Meiβner, Bick & Müller, 2012) or to another type of sensory organ like the dorsal cirrus organ.

Large glandular masses were observed in each parapodium of *E. woodwardi* that are probably parapodial glands associated with chaetae. These glands are similar (although smaller) to the gland-associated chaetal complex that is part of the parapodial glandular organs in Spionidae to which Meiβner, Bick & Müller (2012) suggest a secretory activity related to the chaetogenesis and tube construction.

Cuticular pores are mentioned here for the first time in *E. woodwardi*. Their function is likely to be excretory although the techniques used did not reveal the presence of associated glands. Similar pores were already described for *Eunice* by Ehlers (1868), who suggests an excretory function in *L. harassii* because of being connected to subcutaneous glandular masses.

We also confirmed the presence of nephridia in most segments as it happens in many eunicids (Krishnamoorthi, 1963). Arrangement of nephridia and nephridiopores also agrees with that of other Eunicida such as *Dorvillea rubrovittata* (Grube, 1855), *Leodice torquata* (Quatrefages, 1866) (Fage, 1906) and *Onuphis eremita* Audouin & Milne Edwards, 1833 (Krishnamoorthi, 1963) as well as in Chrysopetalidae Ehlers, 1864 (Tzetlin, Dahlgren & Purschke, 2002). These are probably metanephridia because of their shape and position, as previously described for Eunicidae by Goodrich (1945); however, the lumen and cilia of the nephrostoma could not be clearly seen in *E. woodwardi*.

Oocytes of *E. woodwardi* are very similar to those of *P. siciliensis* (see Hofmann, 1974) and, as in the latter, when they reach maturity, the longitudinal musculature in the posterior body region is much reduced and the intestinal epithelium is atrophied. In addition, this posterior region in *E. woodwardi* also lacks some typical elements such as blood vessels and glandular masses associated with parapodial chaetae, which is consistent with a corporal degeneration that is, in turn, directly linked with release of gametes through liberation of posterior body end. In fact, as previously mentioned, most studied specimens lack the posterior body half, and this might be due to a rough handling of the samples and/or that *E. woodwardi* has indeed an epitokous reproduction mode with release of the posterior body end as was reported by Wilson (1991) in other *Eunice* species.

The shape, size and abundance of the gametes observed through HIS in one specimen of *E. woodwardi*, suggests that it is probably a male with gametes in an early stage of maturation and very similar in size and shape to male gametes reported by Ouassas et al. (2015) for *M. sanguinea*, forming groupings (morulae *sensu*Ouassas et al., 2015) and accumulate in the coelomic cavity of posterior half of the body. Gonads could not be studied in our specimens, and it was not possible to assess whether the oogenesis of *E. woodwardi* is extraovarian or intraovarian. However, an extraovarian strategy was reported by Ouassas et al. (2015) for *M. sanguinea*.

### Ciliophoran epibionts

Epibiosis is a widespread phenomenon and marine annelids show a variety of ecological relationships with other organisms, including different symbiotic associations (Martin & Britayev, 1998) and some have been reported as hosts (basibionts) of a large variety of other taxa (Álvarez-Campos et al., 2014). Mikac et al. (2019) states that this association can be considered as ectocommensalism because the annelid gets no harm from the epibiont. The presence of ciliophorans as epibionts on “polychaetes” has been reported previously in Onuphidae (Arias, Anadón & Paxton, 2010), Syllidae (Álvarez-Campos et al., 2014), Ampharetidae (Parapar et al., 2018), Polynoidae and Sigalionidae (Mikac et al., 2019). Ciliophorans have been found in *E. woodwardi* only on branchiae but in other species can be found also in other parts, such as body surface, prostomium, mouth opening, palps, chaetae, parapodial cirri and pygidium. For instance, in the ampharetid *Ampharete santillani* Parapar, Kongsrud, Kongshavn, Alvestad, Aneiros & Moreira, 2018 the ciliophorans were found across the body but particularly on ciliated areas, such as the branchial surface (Parapar et al., 2018).

## Conclusions

Our examination of the holotype and additional material near the type locality confirms that *E. woodwardi* is a valid species and different to *E. vittata. Eunice woodwardi* is distinguished by the non-articulated and non-constricted cephalic appendages, the maxillary formula, the range of branchial distribution, the maximum number of branchial filaments, the presence of an apical mucro in the guard of falciger chaetae blades, and the number of teeth in pectinate chaetae. *Eunice woodwardi* is at least present in the rias of A Coruña and Ferrol, and we suggest that previous reports of *E. vittata* from the NW Iberian Peninsula should be reviewed. The integrative use of different anatomical techniques for the study of the internal anatomy has confirmed previous observations in the genus *Eunice sensu lato* and allowed reporting some previously unknown structures such as the ciliated areas located below ventral cirrus. The presence of ciliophoran epibionts on branchiae is also reported.

## Supplemental Information

10.7717/peerj.13126/supp-1 Supplemental Information 1 Measurements (µm) of dorsal and ventral parapodial cirri of several examined specimens of *Eunice woodwardi*. CH, chaetiger; dc, dorsal cirrus; L, length; vc, ventral cirrus; W, width.

10.7717/peerj.13126/supp-2 Supplemental Information 2 Number of limbate chaetae per chaetiger of several examined specimens of *Eunice woodwardi* and specimens identified as *E. vittata*. Numbers in brackets: (1), (2) and (3), refer to the examined specimen in a sample or vial composed by several specimens. CH, chaetiger.

10.7717/peerj.13126/supp-3 Supplemental Information 3 Number of compound falciger chaetae per chaetiger of several examined specimens of *Eunice woodwardi* and specimens identified as *E. vittata*. Numbers in brackets: (1), (2) and (3), refer to the examined specimen in a sample or vial composed by several specimens. CH, chaetiger.

## Abbreviations

ac: acicula
br: brain
bra1: branchia 1
bra: branchia
bp: big pore
bs: blood sinus
bv: blood vessel
c: cilium
CH: chaetiger
CH1: chaetiger 1
cfb: compound falciger blade
cha: chaetae
cfbg: compound falciger blade guard
cfs: compound falciger shaft
cgp: cuticular glandular pore
cp: ceratophore
cs: ceratostyle
cut: cuticle
dc: dorsal cirrus
df: fringe of discocilia
dbv: dorsal blood vessel
dst: distal tooth
e: eye
fc: falciger chaeta
FESEM: Field Emission Scanning Electron Microscope
fint: fore intestine
g: gland
hint: hind intestine
HIS: Histological Sectioning
ivc: inflated ventral cirrus
L: length
la: lateral antenna
lc: limbate chaeta
LCM: Light Compound Microscopy
ldm: longitudinal dorsal muscles
lvm: longitudinal ventral muscles
m: mucro
ma: median antenna
man: mandibles
max: maxillae
micro-CT: micro-computed X-ray tomography
mint: mid-intestine
mo: mouth
ms: median sulcus
Mx: maxilla
ne: nephridium
neac: neuroaciculae
ng: nerve ganglia
no: nuchal organ
noac: notoaciculae
np: nephridial pore
oes: oesophagus
ooc: oocytes
pa: palp
par: peristomial anterior ring
pc: peristomial cirrus
pec: pectinate chaeta
pcmt: pectinate chaeta marginal teeth
pg: parapodial gland
pha: pharynx
ppr: peristomial posterior ring
pr: prostomium
prt: proximal tooth
sah: subacicular hook
sahg: subacicular hook guard
SEM: Scanning Electron Microscopy
sp: small pore
spn: supra-oesophageal nerve
stm: stomach
vc: ventral cirrus
vlbv: ventral longitudinal blood vessel
vnc: ventral nerve cord
W: width

## Additional Information and Declarations

### Data Availability

The following information was supplied regarding data availability: *Eunice woodwardi*, Media 000392268: Anterior Part: DOI 10.17602/M2/M392268. *Eunice woodwardi*, Media 000392273: Anterior Part: DOI 10.17602/M2/M392273.
