# Supplementary material for: Long forgotten: Eunice woodwardi Baird, 1869 (Annelida, Eunicidae) revisited, with an insight on internal anatomy
Source: PeerJ. 2022 Apr 14;10:e13126. doi: 10.7717/peerj.13126 (PMC9013482; doi:10.7717/peerj.13126)
Supplement: Supplemental Information 1 — CH, chaetiger; dc, dorsal cirrus; L, length; vc, ventral cirrus; W, width. [file peerj-10-13126-s001.docx]

**Table S1:** Measurements (µm) of dorsal and ventral parapodial cirri of several examined specimens of *Eunice woodwardi*. **CH**–chaetiger, **dc**–dorsal cirrus, **L**–length, **vc**–ventral cirrus, **W**–width.

| **Chaetiger** | | **10** | | | | **30** | | | | **50** | | | | **70** | | | |
| --- | --- | --- | --- | --- | --- | --- | --- | --- | --- | --- | --- | --- | --- | --- | --- | --- | --- |
|  |  | **dc** | | **vc** | | **dc** | | **vc** | | **dc** | | **vc** | | **dc** | | **vc** | |
| **Specimens** | Total CH | L | W | L | W | L | W | L | W | L | W | L | W | L | W | L | W |
| ZH 1863.8.19.13 (Holotype) | 59 | 100 | 125 | 340 | 200 | 1025 | 150 | 375 | 187 | 775 | 75 | 375 | 125 | – | – | – | – |
| MNCN 16.01/19145 | 57 | 650 | 90 | 270 | 175 | 500 | 40 | 220 | 75 | 400 | 30 | 150 | 75 | – | – | – | – |
| MNCN 16.01/19169 | 112 | 925 | 150 | 310 | 180 | 900 | 100 | 300 | 140 | 650 | 125 | 275 | 150 | 800 | 100 | 400 | 130 |
