# Supplementary material for: Long forgotten: Eunice woodwardi Baird, 1869 (Annelida, Eunicidae) revisited, with an insight on internal anatomy
Source: PeerJ. 2022 Apr 14;10:e13126. doi: 10.7717/peerj.13126 (PMC9013482; doi:10.7717/peerj.13126)
Supplement: Supplemental Information 3 — Numbers in brackets: (1), (2) and (3), refer to the examined specimen in a sample or vial composed by several specimens. CH, chaetiger. [file peerj-10-13126-s003.docx]

**Table S3:** Number of compound falciger chaetae per chaetiger of several examined specimens of *Eunice woodwardi* and ­specimens identified as *E. vittata*. Numbers in brackets: (1), (2) and (3), refer to the examined specimen in a sample or vial composed by several specimens. **CH**–chaetiger.

|  | **Chaetiger** | | | | | | | | | | | | | | | |
| --- | --- | --- | --- | --- | --- | --- | --- | --- | --- | --- | --- | --- | --- | --- | --- | --- |
| **Species** | **Total CH** | **1** | **3** | **5** | **10** | **15** | **20** | **25** | **30** | **40** | **50** | **60** | **70** | **80** | **90** | **100** |
| ***Eunice woodwardi*** |  |  |  |  |  |  |  |  |  |  |  |  |  |  |  |  |
| ZH 1863.8.19.13 (Holotype) | 59 | 5 | 15 | 17 | 20 | 16 | 13 | 8 | 10 | 12 | 12 | – | – | – | – | – |
| MNCN 16.01/19145 | 57 | – | 10 | 16 | 11 | 11 | 8 | 8 | 7 | 6 | 4 | – | – | – | – | – |
| MNCN 16.01/19154 | 97 | 5 | 9 | 15 | 14 | 12 | 11 | 9 | 8 | 6 | 7 | 5 | 6 | 5 | 3 | – |
| MNCN 16.01/19159 | 102 | 5 | 11 | 16 | 13 | 13 | 12 | 18 | 14 | 7 | 8 | 8 | 9 | 10 | 6 | 4 |
| MNCN 16.01/19169 | 112 | 7 | 17 | 20 | 20 | 23 | 15 | 20 | 11 | 12 | 11 | 11 | 9 | 9 | 8 | 5 |
| ***Eunice vittata*** |  |  |  |  |  |  |  |  |  |  |  |  |  |  |  |  |
| MNHW (1) | 84 | – | 20 | 23 | 15 | 17 | 12 | 9 | 8 | 8 | 9 | 10 | 8 | 4 | – | – |
| MNHW (2) | 80 | 6 | 16 | 14 | 11 | 10 | 12 | 9 | 9 | 8 | 7 | 6 | 7 | 6 | – | – |
| ZMH-V 12932 (1) | 93 | 4 | 16 | 25 | 24 | 22 | 20 | 11 | 8 | 9 | 11 | 7 | 8 | 6 | 7 | – |
| ZMH-V 12932 (2) | 107 | 3 | 14 | 18 | 18 | 15 | 11 | 10 | 11 | 8 | 7 | 10 | 7 | 7 | 6 | 4 |
| ZMH-V 12932 (3) | 82 | 7 | 18 | 25 | 24 | 19 | 18 | 17 | 15 | 10 | 9 | 6 | 6 | 9 | – | – |
| ZMH-P 14276 (1) | 43 | – | 20 | 19 | 14 | 12 | 9 | 8 | 5 | 8 | – | – | – | – | – | – |
| ZMH-P 14276 (2) | 50 | 4 | 13 | 15 | 8 | 7 | 6 | 4 | 5 | 4 | 4 | – | – | – | – | – |
| MNCN 16.01/2677 | 45 | 6 | 7 | 7 | 10 | 10 | 8 | 8 | 8 | 6 | – | – | – | – | – | – |
| MNCN 16.01/2723 (1) | 107 | 5 | 10 | 13 | 10 | 9 | 8 | 8 | 8 | 6 | 5 | 5 | 5 | 4 | 5 | 3 |
| MNCN 16.01/2723 (2) | 110 | 4 | 6 | 8 | 8 | 9 | 4 | 5 | 4 | 4 | 3 | 3 | 3 | 4 | 4 | 3 |
| SPR04-03 | 59 | 7 | 6 | 11 | 12 | 7 | 6 | 6 | 4 | 5 | 5 | – | – | – | – | – |
